# Supplementary material for: Interview study exploring how global health partnership principles are enacted and recommendations for practice
Source: BMJ Open. 2024 Jun 11;14(6):e076475. doi: 10.1136/bmjopen-2023-076475 (PMC11168132; doi:10.1136/bmjopen-2023-076475)
Supplement: Supplementary data [file bmjopen-2023-076475supp001.pdf]

COREQ (COnsolidated criteria for REporting Qualitative research) Checklist

A checklist of items that should be included in reports of qualitative research. You must report the page number in your manuscript where you consider each of the items listed in this checklist. If you have not included this information, either revise your manuscript accordingly before submitting or note N/A.

Topic	Item No.	Guide Questions/Description	Reported on Page No.
**Domain 1: Research team and reflexivity**			
Personal characteristics			
Interviewer/facilitator	1	Which author/s conducted the interview or focus group?	8
Credentials	2	What were the researcher's credentials? E.g. PhD, MD	n/a
Occupation	3	What was their occupation at the time of the study?	n/a
Gender	4	Was the researcher male or female?	n/a
Experience and training	5	What experience or training did the researcher have?	n/a
Relationship with participants			
Relationship established	6	Was a relationship established prior to study commencement?	8
Participant knowledge of the interviewer	7	What did the participants know about the researcher? e.g. personal goals, reasons for doing the research	n/a
Interviewer characteristics	8	What characteristics were reported about the inter viewer/facilitator? e.g. Bias, assumptions, reasons and interests in the research topic	n/a
**Domain 2: Study design**			
Theoretical framework			
Methodological orientation and Theory	9	What methodological orientation was stated to underpin the study? e.g. grounded theory, discourse analysis, ethnography, phenomenology, content analysis	8
Participant selection			
Sampling	10	How were participants selected? e.g. purposive, convenience, consecutive, snowball	8
Method of approach	11	How were participants approached? e.g. face-to-face, telephone, mail, email	8
Sample size	12	How many participants were in the study?	9
Non-participation	13	How many people refused to participate or dropped out? Reasons?	n/a
Setting			
Setting of data collection	14	Where was the data collected? e.g. home, clinic, workplace	8
Presence of non-participants	15	Was anyone else present besides the participants and researchers?	8
Description of sample	16	What are the important characteristics of the sample? e.g. demographic data, date	8
Data collection			
Interview guide	17	Were questions, prompts, guides provided by the authors? Was it pilot tested?	8
Repeat interviews	18	Were repeat inter views carried out? If yes, how many?	n/a
Audio/visual recording	19	Did the research use audio or visual recording to collect the data?	8
Field notes	20	Were field notes made during and/or after the inter view or focus group?	8
Duration	21	What was the duration of the inter views or focus group?	8
Data saturation	22	Was data saturation discussed?	8
Transcripts returned	23	Were transcripts returned to participants for comment and/or	n/a

Topic	Item No.	Guide Questions/Description	Reported on Page No.
		correction?	
**Domain 3: analysis and findings**			
Data analysis			
Number of data coders	24	How many data coders coded the data?	8-9
Description of the coding tree	25	Did authors provide a description of the coding tree?	n/a
Derivation of themes	26	Were themes identified in advance or derived from the data?	9
Software	27	What software, if applicable, was used to manage the data?	8-9
Participant checking	28	Did participants provide feedback on the findings?	n/a
Reporting			
Quotations presented	29	Were participant quotations presented to illustrate the themes/findings? Was each quotation identified? e.g. participant number	9-18
Data and findings consistent	30	Was there consistency between the data presented and the findings?	9-18
Clarity of major themes	31	Were major themes clearly presented in the findings?	9-18
Clarity of minor themes	32	Is there a description of diverse cases or discussion of minor themes?	9-18

Developed from: Tong A, Sainsbury P, Craig J. Consolidated criteria for reporting qualitative research (COREQ): a 32-item checklist for interviews and focus groups. *International Journal for Quality in Health Care*. 2007. Volume 19, Number 6: pp. 349 – 357

Once you have completed this checklist, please save a copy and upload it as part of your submission. DO NOT include this checklist as part of the main manuscript document. It must be uploaded as a separate file.

MANCHES 182

The University of Ma

Understanding Principles of Partnerships for Commonwealth Antimicrobial Stewardship Schemes

Semi-structured interview schedule

Introduction

Thank you for your time in taking part today. We are interested in your perspective regarding the delivery of key principles of partnerships and what makes health partnerships successful. Just to reiterate there is no right or wrong answer and we are simply interested in your opinion. The interview today and the data generated in the form of transcript will be kept within the research team and any identifiable information or experiences shared will be fully anonymised. No identifiable details will be shared and only general points will be fed back to THET. Do you have any questions?

Before we start, let me explain a bit about what we are going to do, because it may be a little different from what you have done in the past. This will be what's called an 'appreciative interview'. I will be asking you questions about when you have experienced things working at their best and what made it happen that way, because the more we know what makes things work well, the more we can use these successful elements in the future. I won't be asking about problems or searching for ways to fix them. Instead, I would like to hear about what is working well, and the factors that make things work well. Do you have any questions?

Questions

Can you tell me a little bit about the health partnership you are/were involved in?
Probe: Project, aims of project, countries involved.

- Does you project or previous project explore an aspects of behaviour change for antimicrobial stewardship?
  - Probe: How is this being measured, how is this being addressed?

- What behavioural strategies have you found work well in your partnerships?
  - Probe: acceptability

What's really important about this experience? What do you value most about it?

Thinking about the partnership aspects of your work, what do you think has gone well?

What key strengths do you think have been important in this work?

What have you or your partners done well to try to make your partnership strategic?
Probe: shared vision, long-term aims, agreed priorities

What have you or your partners done well to make you partnership aligned to national plans?

What have you or your partners done well to make your partnership meet your target and aims?

What have you or your partners done well to make your partnership reciprocal and respectful?

What have you or your partners done well to make your partnership 'equal' or 'shared'?

What have you or your partners done well to make your partnership organised and accountable?

Probe: Structure, management, transparent, clear

What have you or your partners done well to cultivate trust with partners or other stakeholders?

Where you've experienced changes during your partnership, what have you or your partners done well to make your partnership flexible and innovative in the face of change?

What have you or your partners done well to continue learning together?

What have you or your partners done well to tackle inequalities in your partnership?

Can I ask you to imagine yourself in a year or two years in the future, where the partnership has worked really well.

Probe: What does that look like?
Probe: What has happened?
Probe: How did you make that work?
Probe: What support would you need to achieve this?

Is there anything else you wish to say or discuss today?
