# Supplementary material for: Interview study exploring how global health partnership principles are enacted and recommendations for practice
Source: BMJ Open. 2024 Jun 11;14(6):e076475. doi: 10.1136/bmjopen-2023-076475 (PMC11168132; doi:10.1136/bmjopen-2023-076475)
Supplement: Author's manuscript [file bmjopen-2023-076475.draft_revisions.pdf]

BMJ Open

BMJ Open is committed to open peer review. As part of this commitment we make the peer review history of every article we publish publicly available.

When an article is published we post the peer reviewers' comments and the authors' responses online. We also post the versions of the paper that were used during peer review. These are the versions that the peer review comments apply to.

The versions of the paper that follow are the versions that were submitted during the peer review process. They are not the versions of record or the final published versions. They should not be cited or distributed as the published version of this manuscript.

BMJ Open is an open access journal and the full, final, typeset and author-corrected version of record of the manuscript is available on our site with no access controls, subscription charges or pay-per-view fees (http://bmjopen.bmj.com).

If you have any questions on BMJ Open's open peer review process please email info.bmjopen@bmj.com

BMJ Open

An interview study exploring how global health partnership principles are enacted, what challenges are faced and recommendations for practice

Journal:	*BMJ Open*
Manuscript ID	bmjopen-2023-076475
Article Type:	Original research
Date Submitted by the Author:	08-Jun-2023
Complete List of Authors:	Turner, Rebecca; The University of Manchester, Division of Psychology and Mental Health<br>Hart, Jo; University of Manchester, Division of Medical Education<br>Carr, Natalie; The University of Manchester<br>Bull, Eleanor; The University of Manchester, Division of Medical Education<br>Fraser, Jessica; Tropical Health and Education Trust (THET)<br>Byrne-Davis, Lucie; The University of Manchester, Division of Medical Education
Keywords:	Health policy < HEALTH SERVICES ADMINISTRATION & MANAGEMENT, Health Education, International health services < HEALTH SERVICES ADMINISTRATION & MANAGEMENT

SCHOLARONE™
Manuscripts

I, the Submitting Author has the right to grant and does grant on behalf of all authors of the Work (as defined in the below author licence), an exclusive licence and/or a non-exclusive licence for contributions from authors who are: i) UK Crown employees; ii) where BMJ has agreed a CC-BY licence shall apply, and/or iii) in accordance with the terms applicable for US Federal Government officers or employees acting as part of their official duties; on a worldwide, perpetual, irrevocable, royalty-free basis to BMJ Publishing Group Ltd ("BMJ") its licensees and where the relevant Journal is co-owned by BMJ to the co-owners of the Journal, to publish the Work in this journal and any other BMJ products and to exploit all rights, as set out in our licence.

The Submitting Author accepts and understands that any supply made under these terms is made by BMJ to the Submitting Author unless you are acting as an employee on behalf of your employer or a postgraduate student of an affiliated institution which is paying any applicable article publishing charge ("APC") for Open Access articles. Where the Submitting Author wishes to make the Work available on an Open Access basis (and intends to pay the relevant APC), the terms of reuse of such Open Access shall be governed by a Creative Commons licence – details of these licences and which Creative Commons licence will apply to this Work are set out in our licence referred to above.

Other than as permitted in any relevant BMJ Author's Self Archiving Policies, I confirm this Work has not been accepted for publication elsewhere, is not being considered for publication elsewhere and does not duplicate material already published. I confirm all authors consent to publication of this Work and authorise the granting of this licence.

For peer review only

An interview study exploring how global health partnership principles are enacted, what challenges are faced and recommendations for practice

Turner, R.R., Hart, J., Carr, N., Bull, E., Fraser, J. and Byrne-Davis, L.

Dr Rebecca Turner (RRT)
First and corresponding author: (rebecca.turner@manchester.ac.uk)
Division of Psychology and Mental Health, Faculty of Biology, Medicine & Health, the University of Manchester, Manchester, M13 9PL, UK

Professor Jo Hart (JH)
Second Author
Division of Medical Education, Faculty of Biology, Medicine & Health, the University of Manchester, Manchester, M13 9PG, UK

Natalie Carr (NC)
Third author
Division of Medical Education, Faculty of Biology, Medicine & Health, the University of Manchester, Manchester, UK

Dr Eleanor Bull (EB)
Fourth author
Division of Medical Education, Faculty of Biology, Medicine & Health, the University of Manchester, Manchester, M13 9PG, UK

Jessica Fraser (JF)
Fifth author
The Tropical Health and Education Trust, 3rd Floor, 86-90 Paul Street, London, EC2A 4NE, UK

Professor Lucie Byrne-Davis (LBD)
Senior and last author
Division of Medical Education, Faculty of Biology, Medicine & Health, the University of Manchester, Manchester, M13 9PG, UK

For peer review only

For peer review only - http://bmjopen.bmj.com/site/about/guidelines.xhtml

Data availability statement:

The data underlying this article will be shared on reasonable request to the corresponding author.

Funding:

This project was part of the Commonwealth Partnerships for Antimicrobial Stewardship (CwPAMS), managed by the Tropical Health and Education Trust (THET) and Commonwealth Pharmacists Association (CPA). CwPAMS is funded by the UK Department of Health and Social Care's Fleming Fund using UK aid. The views expressed in this publication are those of the authors and not necessarily those of the UK Department of Health and Social Care, the NHS, the represented NHS Trusts, CPA, or THET.

Conflicts of Interest:

Author JF works for the Tropical Health and Education Trust. No other conflicts of interest to declare.

For peer review only

For peer review only - http://bmjopen.bmj.com/site/about/guidelines.xhtml

Abstract

Background

Effective global health partnerships (HPs) can strengthen and improve health and healthcare systems across the world, however establishing and maintaining effective HPs can be challenging. Principles of Partnerships have been developed to improve the quality and effectiveness of HPs. It is unclear how principles are enacted in practice and current research exploring what makes a global HP successful has not always included the voices of Low-to-Middle Income Country (LMIC) partners. This study aimed to explore how THET's nine Principles of Partnership are implemented in practice, from the points of view of partners from LMICs and High-Income Countries (HICs), to help improve partnerships' quality and sustainability of HPs.

Methods

People who had been a part of previous and/or ongoing HPs, were interviewed virtually. Participants were recruited via purposeful sampling and interviews were conducted using an appreciative inquiry approach. Audio recordings were transcribed and deductive framework analysis was conducted.

Results

Thirteen participants from eight HPs were interviewed. Six participants were based in the LMIC partner, with seven based in the HIC partner. Key findings identified strategies that enacted 'successful' and 'effective' partnerships within the Principles of Partnerships. These included practical techniques such as hiring a project manager, managing expectations and openly sharing information about the team's expertise and aspirations. Other strategies included the importance of consulting behavioural science to ensure the partnerships consider longevity and sustainability of the HP.

Discussion

Core principles to effective partnerships do not work in isolation of each other; they are intertwined and are complimentary to support equitable HPs. Good communication and relationships built on trust which allow all partners to contribute equally throughout the project are core foundations for sustainable HPs. Recommendations for established and future partnerships include embedding behavioural scientists/psychologists to support change to improve the quality and sustainability of HPs.

For peer review only - http://bmjopen.bmj.com/site/about/guidelines.xhtml

What is already known on this topic

- Health partnerships are a central component to global health, to build capacity and improve health and healthcare, but establishing and maintaining successful and effective health partnerships can be challenging.
- Principles of health partnerships have been established by organisations including the Tropical Health and Education Trust to support the development and maintenance of health partnerships.

What this study adds

- An understanding of how principles of health partnerships can be enacted in from the perspective or individuals from both the Low-to-Middle Income Countries and High Income Countries within health partnerships.
- The study also highlights the role of behavioural scientists and applied psychologists to support partnerships teams to think about long-term implementation and behaviour change to ensure the partnership is sustainable.

How this study might affect research, practice or policy

- Exploring how partnership principles work in practice can influence future policy and practice in establishing and maintaining successful health partnerships.

For peer review only

For peer review only - http://bmjopen.bmj.com/site/about/guidelines.xhtml

Background

Health partnerships (HPs) are a way to improve health and healthcare across the world via the exchange of knowledge and skills, building capacity and co-developing interventions between individuals and organisations from different countries (1). Global HPs can be defined as long-term established collaborations between high-income countries (HICs) such as the UK, and low to middle-income Countries (LMICs) that work together to achieve a common goal (2). The long-term aims of HPs vary greatly and previous examples have addressed antibiotic use in healthcare settings (3), helped prioritise mental wellbeing in communities, as well as enabling educating and training of workforces to further build capacity (4, 5).

Establishing successful HPs is a complex intervention in itself. Whilst HPs often work towards a common aim, establishing a successful partnership where teams work effectively, collaboratively, and respectfully is challenging. Global HPs encompass a complex intersection of individual motivations, different professional groups, diverse funding opportunities, and vast health and social inequities that continue within and between countries across the world (6). A growing body of literature has started to explore the key determinants to understanding what makes a HP successful or not. A recent evidence synthesis highlighted commonalities within global HPs (6). Authors identified professional relationships as important for HPs, however many HPs reported failed to address equity within their partnerships. For example, some did not allow all partners to actively contribute to discussions, highlighting power dynamics still exist within global HPs (7). Another challenge within HPs is the issue of colonialism. This is due to stakeholders from HICs driving projects based on their own priorities, leading them to impart their 'superior' Western knowledge on to health workers in LMICs (8). This can lead to a hierarchy, with HICs often referred to as the 'donors', and the hosting country known as the 'recipient' leading to further issues between the partners (9).

Organisations such as the Tropical Health and Education Trust (THET) and the World Health Organisation (WHO) are fundamental organisations in global HPs. THET manages UK aid funding to support access to healthcare across thew world (1). Examples of these include the HP scheme 'Commonwealth Partnershipsfor Antimicrobial Stewardship' (CwPAMS), where HPs aim to address antimicrobial resistance (AMR) challenges in LMICs and in the UK through bidirectional learning (10). THET have developed key principles for HPs to improve the quality and effectiveness of global HPs. These are in the form of nine Principles of Partnership, please see Table 1. One study utilised the THET eight Principles of Partnership (the ninth principle has since been added) as a framework to review a global partnership between the Ministry of Health in Nepal, a healthcare provider and University in California (11). Key findings included a need for efficient working practices to minimise the cost of integration, and an attempt to prioritise the national and host institution's goals while concurrently endeavouring to develop the careers of global health workers.

For peer review only - http://bmjopen.bmj.com/site/about/guidelines.xhtml

Table 1: Definitions of the Tropic Health and Education Trust nine partnership principles

Principles	Definition as provided by THET
Harmonised and aligned with national plans	Health partnerships' work is consistent with local and national plans and complements the activities of other development partners.
Effective and sustainable	Health partnerships operate in a way that delivers high-quality projects that meet targets and achieves long term results.
Strategic	Health partnerships have a shared vision, have long-term aims and measurable plans for achieving them and work within a jointly-agreed framework of priorities and direction.
Respectful and reciprocal	Health partnerships listen to one another and plan, implement and learn together.
Organised and accountable	Health partnerships are well-structured, well-managed and efficient and have clear and transparent decision making processes.
Responsible and build trust with partners	Health partnerships conduct their activities with integrity and cultivate trust in their interactions with stakeholders.
Flexible, resourceful and innovative	Health partnerships proactively adapt and respond to altered circumstances and embrace change.
Committed to joint learning	Health partnerships monitor, evaluate and reflect on their activities and results, articulate lessons learned and share knowledge with others.
Embed equity and inclusion	Health Partnerships consider unequal power relations and inequalities experienced by individuals as a result of their social identities and conduct GESI activities and analysis to ensure GESI is mainstreamed into organizations, programmes, interventions and activities.

1. The Tropical Health and Education Trust. The Tropical Health and Education Trust: Principles of Partnership [Available from: https://www.thet.org/principles-of-partnership/.

Further research is required to explore how THET's principles of partnership, including the new ninth principle *(Embed equity and inclusion)* are enacted within HPs from both LMIC and HIC partners. There is a lack of practical and equitable understanding and support for future healthcare workers and researchers of how HPs can be established and maintained. It is also unclear what exactly makes these HPs work successfully together, if there is evidence about which of these principles are specifically important for change and how they are enacted. By gaining further understanding about what makes HPs work well in practice, we can replicate key elements in future HPs to support effective working and provide practical examples.

The principles of HPs as developed by THET (7) will be explored in Commonwealth Partnerships for Antimicrobial Stewardship (CwPAMS) HPs as part of this study, focusing upon these HPs within one clinical area will act as an exemplar focus. Whilst there are many key organisations within global health, who promote strategies to encourage successful HPs,

For peer review only - http://bmjopen.bmj.com/site/about/guidelines.xhtml

THET is one of the major organisations in HP work in the UK. The aim of this study was to explore and understand how the principles of partnerships are enacted within HPs, to centre the voices of LMIC partners and to provide practical recommendations for current and future partnerships.

Keywords

Health Partnerships, Global Health, Healthcare, Healthcare professionals, Interviews, Qualitative research, Appreciative Inquiry

For peer review only

For peer review only - http://bmjopen.bmj.com/site/about/guidelines.xhtml

Methods

Study design

This was a qualitative, semi-structured interview study, guided by criteria for reporting qualitative research COREQ (12), see Figure 1. The topic guide explored how the key partnership principles advocated by THET (7) were enacted. Appreciative Inquiry (AI) (13) was adopted during the design of the interview questions and by the interviewers throughout the interviews. An appreciative inquiry approach was used to encourage people to talk explicitly about what worked well, what strengths people displayed and how they imagine a successful partnership could be further established (14).

Participants

This context was selected as the partnerships were all based within the same clinical area (antimicrobial stewardship) and a handful of geographical areas. Due to the specific aim of the study, studying a relatively homogenous group of HPs within one clinical area and using a specific framework to focus the study, purposive sampling was used (15). Participants consisted of individuals who were involved, or had previously been involved in a global HP, specifically in HPs exploring antibiotic use in Commonwealth countries.

Recruitment

An initial recruitment email was sent to all potential participants from a representative at THET, asking interested participants to express an interest in the study. Potentially interested participants agreed for their contact details to be passed onto a member of the research team. An email was then sent to these participants with a participant information sheet outlining the purpose of the study, what procedures would take place, data protection and confidentiality, as well as relevant contact information. Willing volunteers responded to the email and interview dates were organised. Informed consent was gained from participants at the start of each interview and recorded, with the interviewer reading out each statement on the consent form, and participants providing their consent.

Data collection

Interviews were conducted over Zoom or MS Teams by RT or NC, who had no prior relationship to the interviewees. Interviewees were encouraged to take part in a confidential space. Interviews followed a semi-structured interview schedule (see Figure 2) as well as probed and follow-up questions based on participant's answers. Interviews lasted approximately 45-75 minutes. Interviews were recorded and transcribed using the auto-transcription tool via Zoom or MS Teams. Transcriptions were checked for accuracy, adjusted to intelligent verbatim transcriptions, and anonymised by redacting any information about people, places or organisations. Transcripts were then uploaded into NVIVO 12 Version 12.5.0.815. Field notes were not used. Sample size was determined by the specific narrow aim of the study and homogeneity of the group (15) and in line with pragmatically with recruitment constraints.

Data analysis

Data was analysed using a deductive framework approach (16), using the nine principles of partnerships developed by THET as a framework (7). RT initially familiarised themselves with

For peer review only - http://bmjopen.bmj.com/site/about/guidelines.xhtml

the data by listening to the audio tapes and re-reading the transcripts. Any key ideas or thoughts were listed at this stage. The principles of partnerships were used as a framework using a coding manual. The framework was applied to each of the transcript using NVIVO 12 Version 12.5.0.815, coding each significant piece of test into the framework. Where text related to more than one principle, it was coded to both. Once all the transcripts were coded, LBD, JH and EB checked 100% of the data and reviewed the data in terms of their significance in relation to the particular framework domain. Data was then summarised and differences and similiarities were identified between participants from the HICs and LMICs and connections between the principles.

Following the framework analysis, recommendations were made to help improve partnerships' quality and sustainability. These recommendations were developed by the research team, based on professional judgement and experience in developing theory-based recommendations for policy and practice.

Ethical approval

This study has received ethical approval from the University of Manchester University Research Ethics Committee 5 (Ref: 2021-12956-21469).

Results

Characteristics of participants

Thirteen individuals from eight partnerships took part in an interview, the majority were pharmacists (69%), and other participants were academics (15%), microbiologists (8%) and project managers (8%). Six participants were based in the LMIC income country partner (Kenya, Ghana or Malawi) and seven participants were based in the HIC partner (UK). Please see Table 2.

Table 2: Characteristics of participants

**Profession**	Pharmacist	8 (62%)
	Microbiologist	1 (8%)
	Project manager	2 (15%)
	Academic	2 (15%)
**Country**	Kenya	2 (15%)
	Ghana	2 (15%)
	Malawi	2 (15%)
	United Kingdom	7 (54%)

Principles of Partnerships

Findings are presented narratively under each partnership principle.

Harmonised and aligned with national plans

To ensure the HPs were aligned with national plans, the teams typically carried out three actions 1) consulted national policy documents, 2) met with local governments and policy makers and 3) adapted guidance from the UK for use in the LMIC.

For peer review only - http://bmjopen.bmj.com/site/about/guidelines.xhtml

National policy documents were consulted at the beginning of the partnership and throughout to ensure the interventions that were developed were in line with the national agendas.

"So right from the beginning, we had copies from the [LMIC] team of their national guidance of their national Antimicrobial Resistance protocol . . . we were all tasked to read them to make sure that what we were devising and doing fitted in quite clearly with the governmental approach that the [LMIC team] was taking." ***(Participant(P)9, Pharmacist, UK)***

Participants from HIC discussed how they would work with local (LMIC) teams to adapt the principles of UK guidelines, which often acted as a starting point for HPs teams. However it was crucial that individuals from the LMIC local context supported the translation of these guidelines into local policies.

"There may be some general principles that are applicable wherever you're working, but you gotta adapt those locally and the people who are gonna help us do that are the people who work closely." ***(P1: Microbiologist, UK)***

Participants from LMICs stated the importance of building upon this by holding regular meeting with key stakeholders within healthcare to further ensure connectivity to national plans and establish relationships with senior people within the healthcare systems.

"We have been involved in different meetings with the Minister of Health. We meet routinely once a quarter or sometimes a month, depending on the need and discuss Antimicrobial Stewardship issues . . . That helps us with our partnership aligned to the national strategy in the country." ***(P11: Pharmacist, Malawi)***

Effective and sustainable

Sustaining the HPs for the long-term was a key priority, to continue to build capacity in LMICs but also to keep partnership workers engaged. Whilst it was acknowledged this cannot be guaranteed, organisations and senior leaders were seen as having a key role to play in supporting HPs and partnership staff in the long-term.

"Nothing is fundamentally ever sustainable, but I think the key elements are, we've got fantastic support now from the hospital leadership. The whole hospital has become aware of a significant change on the ward." ***(P5: Academic, UK)***

Creating this much needed support from HPs organisations and leaders was established by showcasing the benefits that HP volunteering brings to individuals such as new skills, which are then translated into their everyday practice.

"I think the institutional support we have from our trust is fantastic and I don't think we could do that without it. So that's certainly a key factor and I think just giving us that in believing in us. I think the institution and organization sees the value of, for example, workforce development, it brings innovation, it brings those kind of benefits that volunteers come back with." ***(P6: Pharmacist, UK)***

For peer review only - http://bmjopen.bmj.com/site/about/guidelines.xhtml

Ensuring the partnership was effective and sustainable also led to the use of behavioural science, whilst individuals in the partnership did not profess to being experts in this field, they found drawing upon the expertise of applied psychologists and behavioural scientists valuable. Drawing upon this field enlighten the partnerships teams and supported them in thinking about long-term implementation and behaviour change, often something they described as not previously doing.

"The whole psychologists and influencing behaviour changes was a game changer for me. I think we're trying to bring in some of those principles with our own antibiotic interventions we're trying to develop locally. I think that recognizing how fundamental that is and how overlooked it is within many aspects for me and my colleagues working in [redacted] . . . I think that if we can get people from The Change Exchange [behavioural science volunteering programme] embedded in all the partnerships that would make it very big difference into the way things are delivered." ***(P1: Microbiologist, UK)***

Participants from LMICs described how it was helpful for colleagues trained in behaviour change to deliver this aspect, as participants understood behaviours needed to change but did not have the expertise to support this.

"You needed to change certain behaviors. So our colleagues introduced the COM-B model, you know, we try to get to what was going on and then after trainings we conducted another survey to see whether there has been any improvement since. Obviously your colleagues from The Change Exchange led the behavioural change aspect because they were the the specialists within behaviour change. That was easy." ***(P14, Pharmacist, Ghana)***

Strategic

Having a long-term vision for the HPs was discussed amongst partnerships, so whilst the projects within the partnership are of a smaller scale, they were always working towards their overall vision and aim. Both HIC and LMIC participants discussed the constant desire to be considering the next step and exploring where the HP could go.

"So it's kind of having that big vision and recognizing that even though what we're doing seems very small scale like you know guideline writing, developing committees that create guidelines, it's all part of that bigger vision. So it's maintaining that and sharing that bigger vision. ***(P1: Microbiologist, UK)***

"We're always talking. We had a WhatsApp group. We're always discussing, even after the meetings when they had gone, you know, to their guest house and all that, we're still on WhatsApp. We're still talking how we can improve on what we are doing" ***(P12: Project manager, Ghana)***

Ensuring health workers were supported within the team to continue to pursue other projects within the overall aim of the HP was important in maintaining an effective and collegiate team, where individual's skills could develop.

"I try to mobilize my colleagues, we need to do more proposals to support the project to run, because what is most important is that there should be activities for people to do, if there

For peer review only - http://bmjopen.bmj.com/site/about/guidelines.xhtml

are not activities . . . People will lose vision. So, once they lose the vision, they will scatter." ***(P3: Pharmacist, Kenya)***

Respectful and reciprocal

Having a historical relationship between the partners and everyone being familiar with each other in the partnership was attributed to success. This also helped the experience to be enjoyable and participants saw their colleagues within the HPs as friends. The formation of friendships helped to maintain respect amongst the HPs.

"I think firstly, it's because we already have that strong partnership. So there's a natural and good sort of healthy working relationship there anyway. We don't just class them as colleagues, we class them as friends." ***(P6: Pharmacist, UK)***

For new HPs where professional and personal relationships had not necessarily developed yet, respect and mutual trust developed from the HP setting tasks and these being completed by both teams. This perception that people are doing what they said they would, helped to build up a foundation of trust within the HP. As being reliable and accountable was viewed as integral for the HPs from both HIC and LMIC participants.

"You know, we make meetings and they attend when they say they're going to, we do. So there's a certain amount of trust that you build up that way." ***(P4: Pharmacist, UK)***

Participants from HIC discuss the how LMIC found it 'refreshing' that HIC colleagues showed respect to colleagues in LMICs by not being forceful in their approach and thinking they know best.

"There was a comment from somebody from the one of the medical personnel in [redacted] that we met almost saying, oh, it's quite refreshing that you're not coming over here and telling us what we should be doing and we're like, well, no, that's not what we're here for, you know." ***(P13, Pharmacist, UK)***

Organised and accountable

Having individuals or small teams who were specifically responsible for ensuring the partnership was organised and kept to specific timelines, such as project managers, were described by both HIC and LMIC participants as vital for ensuring the partnership was delivered in an effective and timely manner. Partnership teams noticed the difference in performance when project managers were appointed in helping to deliver the project and tasks.

"[Project manager] was instrumental in organizing the Zoom meetings, and she has worked all round to ensure everything is going on well in the project." ***(P2: Pharmacist, Kenya)***

"I think we're very lucky with in [redacted] that we've got support from Global Health Partners, which are full time people who work to support parts of activity happening . . . They've been absolutely fabulous and trying to ensure that we stick the timetables". ***(P1: Microbiologist, UK)***

For peer review only - http://bmjopen.bmj.com/site/about/guidelines.xhtml

LMIC participants viewed it as important that project managers/steering committees were balanced and equal.

"So we have what we call the steering committee within our partnership . . . the committee was also based was also constituted visually equal equality. So you've got a balance across board." **(P14: Pharmacist, Ghana)**

HIC participants discussed developing a work plan that was agreed by all, at the beginning of the specific project, was a useful tool to keep the partnership organised and accountable. This work plan also clearly highlighted expertise, roles and expectations, so the professional relationships and workload could be managed.

"We have a document with them which upholds exactly what we set out to achieve and we refresh that and we make sure we review that sort of time and time again, we'll probably be doing some again now post COVID. It definitely I think it shows a firm commitment and not just on a personal level, but actually an institutional commitment, which I think is really helpful in terms of getting the support and resources that you need behind the partnership." **(P6: Pharmacist, UK)**

Responsible and build trust with partners

Building trust within partnerships was described as something that 'took time' and something that occurred due to familiarity and development of friendships. Having a face-to-face connection and being able to spend time outside of work with colleagues helped to further develop trust within the HP was described by HIC participants.

"But having had that time together, you know not just in work, but also in a social context as well . . . you've got that kind of friendship element as well to it, which I think in the longer run is you know what we need in the partnership that sort of camaraderie and banter that comes with having had that sort of face-to-face interaction I think is important." **(P13: Pharmacist, UK)**

Whereas colleagues from LMICs talk about building trust from transparency and keeping each other accountable. They describe trust building each time a task has been completed from both LMIC and HIC collagues.

"For the whole project to be successive, each and everyone else have to do their part, so it created that mutual respect where no one feels like they're the ones doing more than the other party . . So over time and seeing the progress that we have been making, I think that helped us to devote that trust and that mutual respect." **(P11: Pharmacist, Malawai)**

Flexible, Resourceful and innovative

Allowing the partnership to be flexible, especially during the COVID-19 pandemic was important to allow everyone to contribute equally. Often things would change during the course of the HP and participants discussed having to think innovatively about how to overcome certain obstacles.

For peer review only - http://bmjopen.bmj.com/site/about/guidelines.xhtml

"We had to have some contigency measures like due to one of them, or an example was when maybe flights were not possible to to be used. We have some contigency plans that we had, whereby one of them was the Zoom meetings." ***(P2, Pharmacist, Kenya)***

"Yeah, and thinking innovatively and I think being flexible as well because it's not always time differences and stuff like that. It's not always easy as it as it seems, but you will make it happen." ***(P6: Pharmacist, UK)***

Regular communication with partners and funders throughout the HP was expected, especially if changes needed to be made due to unexpected delays. This helped the HP continue to work towards their overall aim but within the boundaries of the HP that were could often change quickly. Participants also discussed the importance of meeting regularly as a team, so no one had to make difficult decisions on their own.

"So when things come up, which we have to modify things, we notify each other as partners since we already in good contact then we also notify the Commonwealth Pharmacy Association and they were necessary it's because we got especially we need is to do with the change of our expenditure of money and also things that to do with the change of the protocols or change of the implementation plan of the project because we cannot just hold the goals." ***(P11: Pharmacist, Malawi)***

Committed to joint learning

A core element to HPs was considered to be the continued learning that takes place between both countries. This often involved sharing experiences, tools and supporting each other with other issues outside of the HP.

"The real key is that what it says the partnership, it's not about doing it one way, it's about it's about learning both ways. I think that we've learned so much from my [redacted] team as well. It's very much not being a one way thing." ***(P1: Microbiologist, UK)***

Learning was often perceived as a consequence of the HPs but also encouraged throughout by sharing materials or mentoring people. This often went beyond the boundaries of the HPs to support people with their careers or preparation for similar issues such as the COVID-19 pandemic.

"I suppose, behind what we're doing, you know, is trying to say we're here to support, you know, because we've done this ourselves locally, it's trying to support you in doing the same thing, you know, in your context." ***(P13: Pharmacist, UK)***

Embed equity and inclusion

Ensuring the partnership foundations were based on equality, diversity and inclusion was considered a fundamental aspect to establishing the HP. This was sometimes formally monitored, with formal documents developed to ensure equality. These documents were signed when joining the HP, with consequences if breached. These ideas and formalisation of ideas often came from the LMIC participants.

For peer review only - http://bmjopen.bmj.com/site/about/guidelines.xhtml

"I think still based on the grounds of equality and diversity, we ensure, that's there's mutual respect and not just ensuring this. We've got documents that's back, whatever we do and there are sanctions for anyone that goes against the sort of offense, another party, so they, are laid down procedures to ensure that things are streamlined and properly formalized. Every member literally signed onto it. So I mean youv'e consented to abide by the rules, you go against it the loss would be with you." ***(P14: Pharmacist, Ghana)***

The focus and fundamental principle of equality appeared to drive flexibility within the partnership, by allowing the ability to be flexible and considerate of others.

"There's been that flexibility that has really helped, so that everyone is able to contribute equally do their tasks without yeah burdening one person or burdening few people." ***(P8: Project Manager, Malawi)***

A way in which the partnership ensured people were able to contribute equally in a practical sense was by using different platforms to communicate and edit documents.

"I think, using a platform like Google drive has helped us, and the weekly meetings have helped us to be able to share information equally and to share tasks equally. So I think that's basically how we've been doing it." ***(P8: Project Manager, Malawi)***

Recommendations for practice

To ensure this research promotes further effective practice in HPs, recommendations in line with the principles of partnerships have been developed, these are presented in Table 3.

For peer review only - http://bmjopen.bmj.com/site/about/guidelines.xhtml

Table 3: Recommendations for practice to support building a successful partnership

Principle	Example of enactment	Recommendation
Harmonised and aligned with national plans	*"Whatever we do, we try to consult the national policy documents in the national action plan so that at the end of the day, our actions are aligned . . . So you know there's always interconnectivity between whatever we are doing."* ***(P14: Pharmacist, Ghana)***	Guidance from the HIC may be consulted to inform practice in the LMIC as part of the partnership, however this should always be adapted for local use. Engaging with local stakeholders around how such guidance may work within the local context is recommended.
Effective and sustainable	*"There was a big Behavioural change, two of them on the team that came and so they helped with the activities . . . They shared with us, some modules and some metrics that we could use as we needed to continue with the project and actually if we were able to get the staff to accept it and to understand."* ***(P12: Project manager, Ghana)***	Experts in behavioural science should be consulted or embedded in HPs multi-disciplinary teams when aiming to address behavioural change and long-term change. These experts can work alongside clinicians to ensure that interventions and evaluations are behaviourally informed and therefore more likely to lead to change.
Strategic	*"So it's maintaining that and sharing that bigger vision."* ***(P1: Microbiologist, UK)***	HPs should focus upon developing a long-term, overarching vision and strategic aim(s). Whilst partnerships may focus upon small projects at a time, it is recommended that the HPs are formed as a partnership, rather than a project team.

For peer review only - http://bmjopen.bmj.com/site/about/guidelines.xhtml

		Showcasing the work within the HPs to individual's employers and other organisations, can help to raise awareness of HPs and the benefits they can bring to everyone involved, including employers. Utilising case studies, writing up papers for publication and disseminating information is recommended.
Respectful and reciprocal	*"The relationships were being built on kind of basic based on kind of mutual respect and friendship really."* ***(P1: Microbiologist, UK)***	When setting up HPs, it is important to recruit individuals who have the same values and vision for the partnership. Carrying out an informal interview with individuals to get to know them and understand their experience and values is recommended.
Organised and accountable	*"The massive gear change was halfway through the project. We had the project manager appointed."* ***(P13: Pharmacist, UK)***	Appointing a project manager and/or steering committee with individuals is recommended at the beginning of the project. It is important that these individuals have aligned values and help to develop the overall aims and objectives of the partnership.<br><br>Regular communication via platforms such as shared drives or messenger platforms which are acceptable for the HPs team is recommended. This communication should be set out in a communication plan, so everyone in the HPs is aware of when communication is expected but also how this communication with happen.
Responsible and build trust with partners	*"I think it's been built up over years. I wouldn't say it was just, you know, job done on day one kind of thing. I definitely think that's been cultivated over*	Whilst participants discussed that HPs can work successfully without face-to-face visits, these are recommended where appropriate, as if the partnership is in its infancy, this can help to build rapport and trust between teams. Face-to-face visits also help teams gain a further understanding of the context, which can help to better develop interventions.

For peer review only - http://bmjopen.bmj.com/site/about/guidelines.xhtml

	years and years." **(P6: Pharmacist, UK)**	
Flexible, resourceful and innovative	*"There's been that flexibility that has really helped, so that everyone is able to contribute equally do their tasks without burdening one person or burdening few people."* **(P8: Project Manager, Malawi)**	It is recommended that teams develop contracts to highlight expectations of the partnership and how individuals should treat others and be treated, this helped to eliminate any problems in ways of working and set boundaries within the partnership. Developing a work-plan with agreed time plan that allows for flexibility is recommended. This should be co-developed and regularly reviewed as a team.
Committed to joint learning	*"It's kind of this partnership of what we can share and the so much we've learned so much they've learned."* **(P9: Pharmacist, UK)**	Setting up a dual-process learning pathway is recommended, where both teams share and learn from each other, this could include visits/placements in both countries and factoring this into the funding and timeline of the project. Sharing CV's at the start of the project can also help individuals in identify mentors or expertise for further professional development.
Embed equity and inclusion	*"We do have a strategy which is gender equity or inclusion which we've decided to and incorporated into the process."* **(P2: Pharmacist, Kenya)**	Ensuring that the HP embeds equity and inclusion throughout their HP is essential. Formalising this in the form of a strategy for the HP is recommended as this can ensure people are treated equally and supported appropriately as individuals. People can held people accountable if such strategies were breached. It also helps to set expectations for people who may join the HP.

For peer review only - http://bmjopen.bmj.com/site/about/guidelines.xhtml

Discussion

This study identified how key principles of HPs are enacted within HPs, specifically within CwPAMS HPs as an exemplar. Key findings of this study highlight the importance of good communication and transparency throughout HPs, the role of behavioural scientists/psychologists in ensuring sustainability of the HP and allowing all involved in the HP to have the opportunity to be heard and contribute fully.

This study supports previous findings, such as difficulties in communication, which include language barriers (17), or variations in language and behaviours amongst cultures (18) can be barriers to successful global HPs. Similar to findings by Jenkins and colleagues (2021), who found relationships that continue to flourish outside of work where friendships form, and shared appropriate language that invokes 'camaraderie' or 'banter' is fundamental in creating trust and transparency when building partnerships. While it may be important to monitor the frequency, type, and effectiveness of communications in order to evaluate partnership relationships (19), this would not take into account communication and relationships that occur outside of work in a social context. However, a communication plan detailing when and how meetings or updates will take place will help to reduce miscommunication between partners, aligning to both the *responsible* and the *respectful and reciprocal* Principles of Partnerships.

Behavioural scientists and psychologists were identified as playing a crucial role in the *Sustainability and effectiveness* of HPs. Previous research has identified the benefits of behavioural science within HPs (20, 21) and carious projects between the UK and Uganda, Sierra Leone, and Mozambique have demonstrated how behavioural scientists can help to strengthen global HPs whilst improving local health systems (4, 5). The Change Exchange (behavioural science volunteer programme) has aimed to bridge this gap by placing behavioural scientists in HPs including the HPs explored in this study (21). As the HPs all rely on behaviour change, whether that's behaviour change of clinical teams or communities to be successful, adding in experts in this area can help improve the effectiveness of HPs.

The COVID-19 pandemic has highlighted the importance of the *flexible*, *resourceful* and *innovative* principle. Partners have been forced to adapt to an ever-changing pandemic, therefore demonstrating openness and flexibility are fundamental to flourishing partnerships. Furthermore the COVID-19 pandemic disrupted in-person HP activities, whilst exascerbating exisiting inequalities in global health, especially in LMICs (22, 23). Teams had to quickly adapt and rely on virtual interactions and approaches within the HPs (2). Research suggests there are differences between what LMIC and HIC partners value in virtual aspects of HPs (2) and differences in how trust is established virtually compared to face-to-face (24). Having face-to-face contact has been associated with increased initial trust, compared to virtual, but over time, levels of trust are comparable between virtual and face-to-face teams (24). Establishing trust was viewed as a central component within the HPs, being able to have that human interation, coupled with each team completing tasks was found to increase trust within the HP.

For peer review only - http://bmjopen.bmj.com/site/about/guidelines.xhtml

Technology and shared drives have enabled individuals to contribute equally to discussions and documents. However, poor internet connections and conflicting daily priorities amongst institutions can cause difficulties in arranging meetings and document sharing between LMICs and HICs (2, 25). Thus, while individuals engaged in global HPs appear *committed to joint learning*, even during a global pandemic, it is important that the partnership considers the technological capacity within the partnership in order to maintain *respect* and *reciprocity* for all members of the partnership (2).

National governments in LMICs often change priorities for their own countries to meet the demands of international partners with their own agenda (26). For participants within this study, following local and national plans ensured partnerships were *harmonised* and *aligned*. Arguably, this gives individuals direction, and will ensure they are working towards the goal of the project without compromising their own governments' guidelines. It was acknowledged that UK guidelines cannot be implemented into LMICs without change; they must be altered to the local community whilst also utilising the skills of behavioural scientists and psychologists.

Critically, many studies have failed to include voices of all partners involved in global HPs, suggesting that while many authors discuss equity, power relations are still prevalent (19, 27). This study attempted to overcome such issues by recruiting participants from both HICs and LMICs. However, this research is not without its limitations, interviews were carried out using an appreciative inquiry approach (13) and whilst this is a strength and allows the research team to explore the common strengths of establishing and working with HPs, the barriers to enacting principles within HPs were less apparent. Further research should further understand how behaviours within partnerships are enacted and what the influences on these behaviours are, to make a partnership 'work'. Another limitation is the interviews were conducted online due to participants being in different parts of the world. Carrying out the interviews online, whilst practically allowed for the research to be carried out, come with limitations as this can impact upon people's ability to be able to take part in the study due to requiring internet access and the appropriate devices.

Conclusion

HPs are complex, with many different interacting parts. The nine Principles of Partnership developed by THET outline key elements to establish and develop a 'successful' partnership that goes beyond a partnership achieving its short-term project goals. Ten practical recommendations are suggested to support future HPs in enacting these principles, which include expectation setting from the inception of HPs, the role of behavioural scientists/psychologists in supporting HPs and continuing to highlight the importance of the work of global HPs. Further work should continue to explore how HPs are established and sustained and how we best to evaluate HPs.

For peer review only - http://bmjopen.bmj.com/site/about/guidelines.xhtml

Contributorship

LBD and JH acquired the funding for this study. LBD, JH, EB and RT conceived the study design. RT and NC collected the data, with recruitment support from JF. RT led the qualitative analysis, with support from LBD, JH and EB. All authors revised the first manuscript draft and read and approved the final manuscript.

Acknowledgements

We would like to thank the study participants for their time and engagement in the study.

Patient and Public Involvement Statement

Patients and/or the public were not involved in the design, or conduct, or reporting, or dissemination plans of our research.

For peer review only

References

1. THET. The Tropical Health and Education Trust Health Partnership Scheme [Available from: https://www.thet.org/our-work/health-partnership-scheme/.
2. Umphrey L, Paasi G, Windsor W, Abongo G, Evert J, Haq H, et al. Perceived roles, benefits and barriers of virtual global health partnership initiatives: a cross-sectional exploratory study. Global Health Research and Policy. 2022;7(1):11.
3. Sneddon J, Thompson W, Kpobi LN, Ade DA, Sefah IA, Afriyie D, et al. Exploring the Use of Antibiotics for Dental Patients in a Middle-Income Country: Interviews with Clinicians in Two Ghanaian Hospitals. Antibiotics. 2022;11(8):1081.
4. Byrne-Davis L, Bull ER, Burton A, Dharni N, Gillison F, Maltinsky W, et al. How behavioural science can contribute to health partnerships: the case of The Change Exchange. Globalization and Health. 2017;13(1):30.
5. Bull ER, Mason C, Junior FD, Santos LV, Scott A, Ademokun D, et al. Developing nurse medication safety training in a health partnership in Mozambique using behavioural science. Global Health. 2017;13(1):45.
6. Plamondon KM, Brisbois B, Dubent L, Larson CP. Assessing how global health partnerships function: an equity-informed critical interpretive synthesis. Globalization and Health. 2021;17(1):73.
7. The Tropical Health and Education Trust. The Tropical Health and Education Trust: Principles of Partnership [Available from: https://www.thet.org/principles-of-partnership/.
8. Kulesa J, Brantuo NA. Barriers to decolonising educational partnerships in global health. BMJ Global Health. 2021;6(11):e006964.
9. Ackers HL, Ackers-Johnson J. Mobile professional voluntarism and international development: Killing me softly?: Springer Nature; 2017.
10. CwPAMS. [cited 2023 16th February]. Available from: https://commonwealthpharmacy.org/cwpams/.
11. Citrin D, Mehanni S, Acharya B, Wong L, Nirola I, Sherchan R, et al. Power, potential, and pitfalls in global health academic partnerships: review and reflections on an approach in Nepal. Glob Health Action. 2017;10(1):1367161.
12. Tong A, Sainsbury P, Craig J. Consolidated criteria for reporting qualitative research (COREQ): a 32-item checklist for interviews and focus groups. International journal for quality in health care. 2007;19(6):349-57.
13. Cooperrider DL, Srivastva, S. Research in organizational change and development 1987.
14. Cooperrider DL, Whitney D, Stavros JM. Appreciative inquiry handbook: For leaders of change, 2nd ed. San Francisco, CA, US: Berrett-Koehler Publishers; 2008. xxix, 454-xxix, p.
15. Malterud K, Siersma VD, Guassora AD. Sample Size in Qualitative Interview Studies:Guided by Information Power. Qualitative Health Research. 2016;26(13):1753-60.
16. Gale NK, Heath G, Cameron E, Rashid S, Redwood S. Using the framework method for the analysis of qualitative data in multi-disciplinary health research. BMC Med Res Methodol. 2013;13:117.
17. Hagen L, Munkhondya B, Myhre K. Similarities and mutual understanding: exchange experiences in Malawi for host and guest students. International Nursing Review. 2009;56(4):476-82.
18. Kokko R. Future nurses' cultural competencies: what are their learning experiences during exchange and studies abroad? A systematic literature review. J Nurs Manag. 2011;19(5):673-82.

For peer review only - http://bmjopen.bmj.com/site/about/guidelines.xhtml

19. Dean L, Njelesani J, Smith H, Bates I. Promoting sustainable research partnerships: a mixed-method evaluation of a United Kingdom–Africa capacity strengthening award scheme. Health Research Policy and Systems. 2015;13(1):81.
20. Turner RR, Reddy SE, Byrne-Davis L, Bull E, & , Hart J. An interview study to explore applied psychologists' experiences of implementing health psychology in global health partnerships: The Change Exchange. . British Journal of Health Psychology. 2023;In Press.
21. Byrne-Davis LMT, Bull ER, Burton A, Dharni N, Gillison F, Maltinsky W, et al. How behavioural science can contribute to health partnerships: the case of The Change Exchange. Globalization and Health. 2017;13(1):30.
22. Karim N, Rybarczyk MM, Jacquet GA, Pousson A, Aluisio AR, Bilal S, et al. COVID-19 pandemic prompts a paradigm shift in global emergency medicine: multidirectional education and remote collaboration. AEM Education and Training. 2021;5(1):79-90.
23. Shamasunder S, Holmes SM, Goronga T, Carrasco H, Katz E, Frankfurter R, et al. COVID-19 reveals weak health systems by design: why we must re-make global health in this historic moment. Global Public Health. 2020;15(7):1083-9.
24. Breuer C, Hüffmeier J, Hibben F, Hertel G. Trust in teams: A taxonomy of perceived trustworthiness factors and risk-taking behaviors in face-to-face and virtual teams. Human Relations. 2019;73(1):3-34.
25. Birch AP, Tuck J, Malata A, Gagnon AJ. Assessing global partnerships in graduate nursing. Nurse Educ Today. 2013;33(11):1288-94.
26. Schriger SH, Binagwaho A, Keetile M, Kerry V, Mubiligi JM, Ramogola-Masire D, et al. Hierarchy of qualities in global health partnerships: a path towards equity and sustainability. BMJ Glob Health. 2021;6(12).
27. Monette EM, McHugh D, Smith MJ, Canas E, Jabo N, Henley P, et al. Informing 'good' global health research partnerships: A scoping review of guiding principles. Global Health Action. 2021;14(1):1892308.

For peer review only

For peer review only - http://bmjopen.bmj.com/site/about/guidelines.xhtml

COREQ (COnsolidated criteria for REporting Qualitative research) Checklist

A checklist of items that should be included in reports of qualitative research. You must report the page number in your manuscript where you consider each of the items listed in this checklist. If you have not included this information, either revise your manuscript accordingly before submitting or note N/A.

Topic	Item No.	Guide Questions/Description	Reported on Page No.
**Domain 1: Research team and reflexivity**			
Personal characteristics			
Interviewer/facilitator	1	Which author/s conducted the interview or focus group?	
Credentials	2	What were the researcher's credentials? E.g. PhD, MD	
Occupation	3	What was their occupation at the time of the study?	
Gender	4	Was the researcher male or female?	
Experience and training	5	What experience or training did the researcher have?	
Relationship with participants			
Relationship established	6	Was a relationship established prior to study commencement?	
Participant knowledge of the interviewer	7	What did the participants know about the researcher? e.g. personal goals, reasons for doing the research	
Interviewer characteristics	8	What characteristics were reported about the inter viewer/facilitator? e.g. Bias, assumptions, reasons and interests in the research topic	
**Domain 2: Study design**			
Theoretical framework			
Methodological orientation and Theory	9	What methodological orientation was stated to underpin the study? e.g. grounded theory, discourse analysis, ethnography, phenomenology, content analysis	
Participant selection			
Sampling	10	How were participants selected? e.g. purposive, convenience, consecutive, snowball	
Method of approach	11	How were participants approached? e.g. face-to-face, telephone, mail, email	
Sample size	12	How many participants were in the study?	
Non-participation	13	How many people refused to participate or dropped out? Reasons?	
Setting			
Setting of data collection	14	Where was the data collected? e.g. home, clinic, workplace	
Presence of non-participants	15	Was anyone else present besides the participants and researchers?	
Description of sample	16	What are the important characteristics of the sample? e.g. demographic data, date	
Data collection			
Interview guide	17	Were questions, prompts, guides provided by the authors? Was it pilot tested?	
Repeat interviews	18	Were repeat inter views carried out? If yes, how many?	
Audio/visual recording	19	Did the research use audio or visual recording to collect the data?	
Field notes	20	Were field notes made during and/or after the inter view or focus group?	
Duration	21	What was the duration of the inter views or focus group?	
Data saturation	22	Was data saturation discussed?	
Transcripts returned	23	Were transcripts returned to participants for comment and/or	

For peer review only

Topic	Item No.	Guide Questions/Description	Reported on Page No.
		correction?	
**Domain 3: analysis and findings**			
Data analysis			
Number of data coders	24	How many data coders coded the data?	
Description of the coding tree	25	Did authors provide a description of the coding tree?	
Derivation of themes	26	Were themes identified in advance or derived from the data?	
Software	27	What software, if applicable, was used to manage the data?	
Participant checking	28	Did participants provide feedback on the findings?	
Reporting			
Quotations presented	29	Were participant quotations presented to illustrate the themes/findings? Was each quotation identified? e.g. participant number	
Data and findings consistent	30	Was there consistency between the data presented and the findings?	
Clarity of major themes	31	Were major themes clearly presented in the findings?	
Clarity of minor themes	32	Is there a description of diverse cases or discussion of minor themes?	

Developed from: Tong A, Sainsbury P, Craig J. Consolidated criteria for reporting qualitative research (COREQ): a 32-item checklist for interviews and focus groups. *International Journal for Quality in Health Care*. 2007. Volume 19, Number 6: pp. 349 – 357

Once you have completed this checklist, please save a copy and upload it as part of your submission. DO NOT include this checklist as part of the main manuscript document. It must be uploaded as a separate file.

For peer review only

Understanding Principles of Partnerships for Commonwealth Antimicrobial Stewardship Schemes

Semi-structured interview schedule

Introduction

Thank you for your time in taking part today. We are interested in your perspective regarding the delivery of key principles of partnerships and what makes health partnerships successful. Just to reiterate there is no right or wrong answer and we are simply interested in your opinion. The interview today and the data generated in the form of transcript will be kept within the research team and any identifiable information or experiences shared will be fully anonymised. No identifiable details will be shared and only general points will be fed back to THET. Do you have any questions?

Before we start, let me explain a bit about what we are going to do, because it may be a little different from what you have done in the past. This will be what's called an 'appreciative interview'. I will be asking you questions about when you have experienced things working at their best and what made it happen that way, because the more we know what makes things work well, the more we can use these successful elements in the future. I won't be asking about problems or searching for ways to fix them. Instead, I would like to hear about what is working well, and the factors that make things work well. Do you have any questions?

Questions

Can you tell me a little bit about the health partnership you are/were involved in?
    Probe: Project, aims of project, countries involved.

- Does you project or previous project explore an aspects of behaviour change for antimicrobial stewardship?
    - Probe: How is this being measured, how is this being addressed?
- What behavioural strategies have you found work well in your partnerships?
    - Probe: acceptability

What's really important about this experience? What do you value most about it?

Thinking about the partnership aspects of your work, what do you think has gone well?

What key strengths do you think have been important in this work?

For peer review only - http://bmjopen.bmj.com/site/about/guidelines.xhtml

What have you or your partners done well to try to make your partnership strategic?

Probe: shared vision, long-term aims, agreed priorities

What have you or your partners done well to make you partnership aligned to national plans?

What have you or your partners done well to make your partnership meet your target and aims?

What have you or your partners done well to make your partnership reciprocal and respectful?

What have you or your partners done well to make your partnership 'equal' or 'shared'?

What have you or your partners done well to make your partnership organised and accountable?

Probe: Structure, management, transparent, clear

What have you or your partners done well to cultivate trust with partners or other stakeholders?

Where you've experienced changes during your partnership, what have you or your partners done well to make your partnership flexible and innovative in the face of change?

What have you or your partners done well to continue learning together?

What have you or your partners done well to tackle inequalities in your partnership?

Can I ask you to imagine yourself in a year or two years in the future, where the partnership has worked really well.

Probe: What does that look like?
Probe: What has happened?
Probe: How did you make that work?
Probe: What support would you need to achieve this?

Is there anything else you wish to say or discuss today?

For peer review only

For peer review only - http://bmjopen.bmj.com/site/about/guidelines.xhtml

BMJ Open

An interview study exploring how global health partnership principles are enacted and recommendations for practice

Journal:	*BMJ Open*
Manuscript ID	bmjopen-2023-076475.R1
Article Type:	Original research
Date Submitted by the Author:	16-Dec-2023
Complete List of Authors:	Turner, Rebecca; The University of Manchester, Division of Psychology and Mental Health<br>Hart, Jo; University of Manchester, Division of Medical Education<br>Carr, Natalie; The University of Manchester<br>Bull, Eleanor; The University of Manchester, Division of Medical Education<br>Fraser, Jessica; Tropical Health and Education Trust (THET)<br>Byrne-Davis, Lucie; The University of Manchester, Division of Medical Education
**Primary Subject Heading**:	Global health
Secondary Subject Heading:	Qualitative research
Keywords:	Health policy < HEALTH SERVICES ADMINISTRATION & MANAGEMENT, Health Education, International health services < HEALTH SERVICES ADMINISTRATION & MANAGEMENT

SCHOLARONE™
Manuscripts

I, the Submitting Author has the right to grant and does grant on behalf of all authors of the Work (as defined in the below author licence), an exclusive licence and/or a non-exclusive licence for contributions from authors who are: i) UK Crown employees; ii) where BMJ has agreed a CC-BY licence shall apply, and/or iii) in accordance with the terms applicable for US Federal Government officers or employees acting as part of their official duties; on a worldwide, perpetual, irrevocable, royalty-free basis to BMJ Publishing Group Ltd ("BMJ") its licensees and where the relevant Journal is co-owned by BMJ to the co-owners of the Journal, to publish the Work in this journal and any other BMJ products and to exploit all rights, as set out in our licence.

The Submitting Author accepts and understands that any supply made under these terms is made by BMJ to the Submitting Author unless you are acting as an employee on behalf of your employer or a postgraduate student of an affiliated institution which is paying any applicable article publishing charge ("APC") for Open Access articles. Where the Submitting Author wishes to make the Work available on an Open Access basis (and intends to pay the relevant APC), the terms of reuse of such Open Access shall be governed by a Creative Commons licence – details of these licences and which Creative Commons licence will apply to this Work are set out in our licence referred to above.

Other than as permitted in any relevant BMJ Author's Self Archiving Policies, I confirm this Work has not been accepted for publication elsewhere, is not being considered for publication elsewhere and does not duplicate material already published. I confirm all authors consent to publication of this Work and authorise the granting of this licence.

For peer review only

An interview study exploring how global health partnership principles are enacted and recommendations for practice

Turner, R.R., Hart, J., Carr, N., Bull, E., Fraser, J. and Byrne-Davis, L.M.T.

Dr Rebecca Turner (RRT)
First author: (rebecca.turner@manchester.ac.uk)
Division of Psychology and Mental Health, Faculty of Biology, Medicine & Health, the University of Manchester, Manchester, M13 9PL, UK

Professor Jo Hart (JH)
Second Author
Division of Medical Education, Faculty of Biology, Medicine & Health, the University of Manchester, Manchester, M13 9PG, UK

Natalie Carr (NC)
Third author
Division of Medical Education, Faculty of Biology, Medicine & Health, the University of Manchester, Manchester, UK

Dr Eleanor Bull (EB)
Fourth author
Division of Medical Education, Faculty of Biology, Medicine & Health, the University of Manchester, Manchester, M13 9PG, UK

Jessica Fraser (JF)
Fifth author
The Tropical Health and Education Trust, 3rd Floor, 86-90 Paul Street, London, EC2A 4NE, UK

Professor Lucie Byrne-Davis (LBD)
Senior, corresponding and last author (lucie.byrne-davis@manchester.ac.uk)
Division of Medical Education, Faculty of Biology, Medicine & Health, the University of Manchester, Manchester, M13 9PG, UK

For peer review only - http://bmjopen.bmj.com/site/about/guidelines.xhtml

Data availability statement:

The data underlying this article will be shared on reasonable request to the corresponding author.

Funding:

This project was part of the Commonwealth Partnerships for Antimicrobial Stewardship (CwPAMS), managed by the Tropical Health and Education Trust (THET) and Commonwealth Pharmacists Association (CPA). CwPAMS is funded by the UK Department of Health and Social Care's Fleming Fund using UK aid. The views expressed in this publication are those of the authors and not necessarily those of the UK Department of Health and Social Care, the NHS, the represented NHS Trusts, CPA, or THET.

Conflicts of Interest:

Author JF works for the Tropical Health and Education Trust. LBD JH and EB have received funding from THET to conduct this research and other studies. No other conflicts of interest to declare.

For peer review only

Abstract

Background

Effective global health partnerships can strengthen and improve health and healthcare systems across the world, however establishing and maintaining effective partnerships can be challenging. Principles of Partnerships have been developed to improve the quality and effectiveness of health partnerships. It is unclear how principles are enacted in practice and current research has not always included the voices of low-and middle- Income country partners. This study aimed to explore how The Tropical Health and Education Trust's nine Principles of Partnership are enacted in practice, from the points of view of partners from low-, middle- and high- income countries, to help improve partnerships' quality and sustainability.

Methods

People who had been a part of previous and/or ongoing health partnerships, were interviewed virtually. Participants were purposefully sampled and interviews were conducted using an appreciative inquiry approach. Audio recordings were transcribed and deductive framework analysis was conducted.

Results

Thirteen participants from eight partnerships were interviewed. Six participants were based in the low- or middle- income countries and seven in the UK. Key findings identified strategies that enacted 'successful' and 'effective' partnerships within the Principles of Partnerships. These included practical techniques such as hiring a project manager, managing expectations and openly sharing information about the team's expertise and aspirations. Other strategies included the importance of consulting behavioural science to ensure the partnerships consider longevity and sustainability of the partnership.

Discussion

Core principles to effective partnerships do not work in isolation of each other; they are intertwined and are complimentary to support equitable partnerships. Good communication and relationships built on trust which allow all partners to contribute equally throughout the project are core foundations for sustainable partnerships. Recommendations for established and future partnerships include embedding behavioural scientists/psychologists to support change to improve the quality and sustainability of health partnerships.

Strengths and limitations of this study

- The participants of this study were all participating in a single scheme focused on antimicrobial stewardship, how they enacted principles of partnership might be different to other participants with different project goals.

For peer review only - http://bmjopen.bmj.com/site/about/guidelines.xhtml

- We took a deductive approach to exploring partnership principles, building on previous work in this area.
- We used appreciative inquiry to structure the interviews; this has the benefit of allowing participants to explore challenges without focusing on the negative, but might also prevent participants from raising failures.

For peer review only

For peer review only - http://bmjopen.bmj.com/site/about/guidelines.xhtml

Background

Health partnerships are a way to improve health and healthcare across the world via the exchange of knowledge and skills, building capacity and co-developing interventions between individuals and organisations from different countries (1). Global health partnerships can be defined as long-term established collaborations between high-income countries (HICs) such as the UK, and low to middle-income Countries (LMICs) that work together to achieve a common goal (2). The long-term aims of health partnerships vary greatly and previous examples have addressed antibiotic use in healthcare settings (3), helped prioritise mental wellbeing in communities, as well as enabling educating and training of workforces to further build capacity (4, 5).

Establishing successful health partnerships is a complex intervention in itself. Whilst they often work towards a common aim, establishing a successful partnership where teams work effectively, collaboratively, and respectfully is challenging. Global health partnerships encompass a complex intersection of individual motivations, different professional groups, diverse funding opportunities, and vast health and social inequities that continue within and between countries across the world (6). A growing body of literature has started to explore the key determinants to understanding what makes a health partnership successful or not. A recent evidence synthesis highlighted commonalities within global health partnerships (6). Authors identified professional relationships as important for partnerships, however many failed to address equity within their partnerships. For example, some did not allow all partners to actively contribute to discussions, highlighting power dynamics still exist within global health partnerships (7). Another challenge within health partnerships is the issue of colonialism. This is due to stakeholders from HICs driving projects based on their own priorities, leading them to impart their 'superior' Western knowledge on to health workers in LMICs (8). This can lead to a hierarchy, with HICs often referred to as the 'donors', and the hosting country known as the 'recipient' (9).

Organisations such as the Tropical Health and Education Trust (THET) and the World Health Organisation (WHO) are fundamental organisations in global health partnerships. THET manages UK aid funding to support access to healthcare across thew world (1). Examples of these include the 'Commonwealth Partnerships for Antimicrobial Stewardship' (CwPAMS) scheme, where health partnerships aim to address antimicrobial resistance challenges in LMICs and in the UK through bidirectional exchange (10). THET have developed key principles for health partnerships to improve their quality and effectiveness. These are in the form of nine Principles of Partnership (see Table 1). One study utilised the THET eight Principles of Partnership (the ninth principle has since been added) as a framework to review a global partnership between the Ministry of Health in Nepal, a healthcare provider and University in California (11). Key findings included a need for efficient working practices to minimise the cost of integration, and an attempt to prioritise the national and host institution's goals while concurrently endeavouring to develop the careers of global health workers.

For peer review only - http://bmjopen.bmj.com/site/about/guidelines.xhtml

Table 1: Definitions of the Tropic Health and Education Trust nine partnership principles

Principles	Definition as provided by THET
Harmonised and aligned with national plans	Health partnerships' work is consistent with local and national plans and complements the activities of other development partners.
Effective and sustainable	Health partnerships operate in a way that delivers high-quality projects that meet targets and achieves long term results.
Strategic	Health partnerships have a shared vision, have long-term aims and measurable plans for achieving them and work within a jointly-agreed framework of priorities and direction.
Respectful and reciprocal	Health partnerships listen to one another and plan, implement and learn together.
Organised and accountable	Health partnerships are well-structured, well-managed and efficient and have clear and transparent decision making processes.
Responsible and build trust with partners	Health partnerships conduct their activities with integrity and cultivate trust in their interactions with stakeholders.
Flexible, resourceful and innovative	Health partnerships proactively adapt and respond to altered circumstances and embrace change.
Committed to joint learning	Health partnerships monitor, evaluate and reflect on their activities and results, articulate lessons learned and share knowledge with others.
Embed equity and inclusion	Health Partnerships consider unequal power relations and inequalities experienced by individuals as a result of their social identities and conduct GESI activities and analysis to ensure GESI is mainstreamed into organizations, programmes, interventions and activities.

1. The Tropical Health and Education Trust. The Tropical Health and Education Trust: Principles of Partnership [Available from: https://www.thet.org/principles-of-partnership/.

Further research is required to explore how THET's principles of partnership, including the new ninth principle *(Embed equity and inclusion)* are enacted within health partnerships from both LMIC and HIC partners. There is a lack of practical and equitable understanding and support for future healthcare workers and researchers of how partnerships can be established and maintained. It is also unclear what exactly makes these partnerships work successfully together, if there is evidence about which of these principles are specifically important for change and how they are enacted. By gaining further understanding about what makes health partnerhips work well in practice, we can replicate key elements in future partnerships to support effective working and provide practical examples.

The principles of health partnerships as developed by THET (7) were explored in Commonwealth Partnerships for Antimicrobial Stewardship (CwPAMS) partnerships as part of this study, focusing upon these health partnerships within one clinical area will act as an

For peer review only - http://bmjopen.bmj.com/site/about/guidelines.xhtml

exemplar focus. Whilst there are many key organisations within global health, who promote strategies to encourage successful partnerships, THET is one of the major organisations in health partnership work in the UK. The aim of this study was to explore and understand how the principles of partnerships are enacted within health partnerships, to centre the voices of LMIC partners and to provide practical recommendations for current and future partnerships.

Keywords
Health Partnerships, Global Health, Healthcare, Healthcare professionals, Interviews, Qualitative research, Appreciative Inquiry

For peer review only

Methods

Study design

This was a qualitative, semi-structured interview study, guided by criteria for reporting qualitative research COREQ (12), included in supplementary materials. The topic guide explored how the key partnership principles advocated by THET (7) were enacted. Appreciative Inquiry (AI) (13) was adopted during the design of the interview questions and by the interviewers throughout the interviews. An appreciative inquiry approach was used to encourage people to talk explicitly about what worked well, what strengths people displayed and how they imagine a successful partnership could be further established (14). After data analysis, recommendations were made to help improve partnerships' quality and sustainability. These recommendations were developed by the research team, based on professional judgement and experience in developing theory-based recommendations for policy and practice.

Research team

RT is a woman and health psychology researcher with an MSc and PhD in health psychology, working as a postdoctoral research associate. JH is a woman, professor of health professional education, an HCPC registered health psychologist, with an MSc and PhD in health psychology. NC is a woman with an MSc in health psychology, currently undertaking a PhD in international health workforce. EB is a woman, an HCPC registered health psychologist, with an MSc and PhD in health psychology, working in public health and the NHS. JF is a woman working as programmes coordinator in an international NGO. She has a Batchelor's degree in international social policy and a masters in global health. LBD is a woman, a professor of health psychology and an HCPC registered health psychologist, with an MSc and PhD in health psychology. EB was embedded in one of the health partnerships and LBD and JH supervised behavioural science volunteers in five of the other health partnerships.

Participants

This context was selected as the partnerships were all based within the same clinical area (antimicrobial stewardship) and a handful of geographical areas. Due to the specific aim of the study, studying a relatively homogenous group of health partnerships within one clinical area and using a specific framework to focus the study, purposive sampling was used (15). Participants consisted of individuals who were involved, or had previously been involved in a global health partnerships exploring antibiotic use in Commonwealth countries. Participants were not paid for participation in this study.

Recruitment

An initial recruitment email was sent to all potential participants from a representative at THET, asking interested participants to express an interest in the study. There were 12 partnerships but the final number of potential participants is unknown to us, as the partnerships themselves vary in terms of how many people are involved at any one time in the partnerships. We estimate that there would be at least four participants per partnership, giving us minimum potential pool of participants of 48. Potentially interested participants agreed for their contact details to be passed onto a member of the research

For peer review only - http://bmjopen.bmj.com/site/about/guidelines.xhtml

team. An email was then sent to these participants with a participant information sheet outlining the purpose of the study, what procedures would take place, data protection and confidentiality, as well as relevant contact information. Willing volunteers responded to the email and interview dates were organised. Informed consent was gained from participants at the start of each interview and recorded, with the interviewer reading out each statement on the consent form, and participants providing their consent. We included all willing participants.

Data collection

Interviews were conducted over Zoom or MS Teams by RT or NC, who had no prior relationship to the interviewees. Interviewees were encouraged to take part in a confidential space. Interviews followed a semi-structured interview schedule (included in supplementary materials) as well as probed and follow-up questions based on participant's answers. Interviews lasted approximately 45-75 minutes. Interviews were recorded and transcribed using the auto-transcription tool via Zoom or MS Teams. Transcriptions were checked for accuracy, adjusted to intelligent verbatim transcriptions, and anonymised by redacting any information about people, places or organisations. Transcripts were then uploaded into NVIVO 12 Version 12.5.0.815. Field notes were not used. Sample size was determined by the specific narrow aim of the study and homogeneity of the group (15) and in line with pragmatically with recruitment constraints.

Data analysis

Data were analysed using a deductive framework approach (16), using the nine principles of partnerships developed by THET as the framework (7). RT initially familiarised themselves with the data by listening to the audio tapes and re-reading the transcripts. Any key ideas or thoughts were listed at this stage. The framework was applied to each of the transcript using NVIVO 12 Version 12.5.0.815, coding each significant piece of test into the framework. Where text related to more than one principle, it was coded to both. Once all the transcripts were coded, LBD, JH and EB checked 100% of the data between them and checked their significance in relation to the particular framework domain. Where there were differences, these were discussed and resolved between that coder and RT. Data were then summarised by RT. RT then continued with the analysis taking a thematic approach, generating themes, identifying differences and similiarities between participants from the HICs and LMICs and connections between the principles. At this point, quotes were selected to demonstrate the breadth and depth of the theme within the framework.

Ethical approval

This study received ethical approval from the University of Manchester University Research Ethics Committee 5 (Ref: 2021-12956-21469).

Results

Characteristics of participants

Thirteen individuals from eight partnerships took part in an interview, the majority were pharmacists (69%), and other participants were academics (15%), microbiologists (8%) and project managers (8%). Six participants were based in the LMIC income country partner

For peer review only - http://bmjopen.bmj.com/site/about/guidelines.xhtml

(Kenya, Ghana or Malawi) and seven participants were based in the HIC partner (UK). Due to the partnerships having a relatively small number of people and our commitment to anonymity, we have included characteristics that are unlikely to de-anonymise the participants (i.e., profession and country) only. Please see Table 2. Participants are identified after each quote by an assigned number e.g., P1.

Table 2: Characteristics of participants (n=13)

**Profession**	Pharmacist	8
	Microbiologist	1
	Project manager	2
	Academic	2
**Country**	Kenya	2
	Ghana	2
	Malawi	2
	United Kingdom	7

Principles of Partnerships

Findings are presented narratively under each partnership principle.

Harmonised and aligned with national plans

To ensure the health partnerships were aligned with national plans, the teams typically carried out three actions 1) consulted national policy documents, 2) met with local governments and policy makers and 3) adapted guidance from the UK for use in the LMIC.

National policy documents were consulted at the beginning of the partnership and throughout to ensure the interventions that were developed were in line with the national agendas.

"So right from the beginning, we had copies from the [LMIC] team of their national guidance of their national Antimicrobial Resistance protocol . . . we were all tasked to read them to make sure that what we were devising and doing fitted in quite clearly with the governmental approach that the [LMIC team] was taking." ***(P9: Pharmacist, UK)***

Participants from HIC discussed how they would work with local (LMIC) teams to adapt the principles of UK guidelines, which often acted as a starting point for teams. However it was crucial that individuals from the LMIC local context supported the translation of these guidelines into local policies.

"There may be some general principles that are applicable wherever you're working, but you gotta adapt those locally and the people who are gonna help us do that are the people who work closely." ***(P1: Microbiologist, UK)***

Participants from LMICs stated the importance of building upon this by holding regular meeting with key stakeholders within healthcare to further ensure connectivity to national plans and establish relationships with senior people within the healthcare systems.

For peer review only - http://bmjopen.bmj.com/site/about/guidelines.xhtml

"We have been involved in different meetings with the Minister of Health. We meet routinely once a quarter or sometimes a month, depending on the need and discuss Antimicrobial Stewardship issues . . . That helps us with our partnership aligned to the national strategy in the country." ***(P11: Pharmacist, Malawi)***

Effective and sustainable

Sustaining the health partnerships for the long-term was a key priority, to continue to build capacity in LMICs but also to keep partnership workers engaged. Whilst it was acknowledged this cannot be guaranteed, organisations and senior leaders were seen as having a key role to play in supporting partners in the long-term.

"Nothing is fundamentally ever sustainable, but I think the key elements are, we've got fantastic support now from the hospital leadership. The whole hospital has become aware of a significant change on the ward." ***(P5: Academic, UK)***

Creating this much needed support from health partnerships organisations and leaders was established by showcasing the benefits that volunteering brings to individuals such as new skills, which are then translated into their everyday practice.

"I think the institutional support we have from our trust is fantastic and I don't think we could do that without it. So that's certainly a key factor and I think just giving us that in believing in us. I think the institution and organization sees the value of, for example, workforce development, it brings innovation, it brings those kind of benefits that volunteers come back with." ***(P6: Pharmacist, UK)***

Ensuring the partnership was effective and sustainable also led to the use of behavioural science, whilst individuals in the partnership did not profess to being experts in this field, they found drawing upon the expertise of applied psychologists and behavioural scientists valuable. Drawing upon this field enlighten the partnerships teams and supported them in thinking about long-term implementation and behaviour change, often something they described as not previously doing.

"The whole psychologists and influencing behaviour changes was a game changer for me. I think we're trying to bring in some of those principles with our own antibiotic interventions we're trying to develop locally. I think that recognizing how fundamental that is and how overlooked it is within many aspects for me and my colleagues working in [redacted] . . . I think that if we can get people from The Change Exchange [behavioural science volunteering programme] embedded in all the partnerships that would make it very big difference into the way things are delivered." ***(P1: Microbiologist, UK)***

Participants from LMICs described how it was helpful for colleagues trained in behaviour change to deliver this aspect, as participants understood behaviours needed to change but did not have the expertise to support this.

"You needed to change certain behaviors. So our colleagues introduced the COM-B model, you know, we try to get to what was going on and then after trainings we conducted another survey to see whether there has been any improvement since. Obviously your

For peer review only - http://bmjopen.bmj.com/site/about/guidelines.xhtml

colleagues from The Change Exchange led the behavioural change aspect because they were the the specialists within behaviour change. That was easy." ***(P14, Pharmacist, Ghana)***

Strategic

Having a long-term vision for the health partnership was discussed amongst partnerships, so whilst the projects within the partnership are of a smaller scale, they were always working towards their overall vision and aim. Both HIC and LMIC participants discussed the constant desire to be considering the next step and exploring where the partnership could go.

"So it's kind of having that big vision and recognizing that even though what we're doing seems very small scale like you know guideline writing, developing committees that create guidelines, it's all part of that bigger vision. So it's maintaining that and sharing that bigger vision. ***(P1: Microbiologist, UK)***

"We're always talking. We had a WhatsApp group. We're always discussing, even after the meetings when they had gone, you know, to their guest house and all that, we're still on WhatsApp. We're still talking how we can improve on what we are doing" ***(P12: Project manager, Ghana)***

Ensuring health workers were supported within the team to continue to pursue other projects within the overall aim of the partnership was important in maintaining an effective and collegiate team, where individual's skills could develop.

"I try to mobilize my colleagues, we need to do more proposals to support the project to run, because what is most important is that there should be activities for people to do, if there are not activities . . . People will lose vision. So, once they lose the vision, they will scatter." ***(P3: Pharmacist, Kenya)***

Respectful and reciprocal

Having a historical relationship between the partners and everyone being familiar with each other in the partnership was attributed to success. This also helped the experience to be enjoyable and participants saw their colleagues as friends. The formation of friendships helped to maintain respect amongst the partners.

"I think firstly, it's because we already have that strong partnership. So there's a natural and good sort of healthy working relationship there anyway. We don't just class them as colleagues, we class them as friends." ***(P6: Pharmacist, UK)***

For new partnerships where professional and personal relationships had not necessarily developed yet, respect and mutual trust developed by setting tasks completed by both teams. This perception that people are doing what they said they would, helped to build up a foundation of trust, as being reliable and accountable was viewed as integral for the partnership from both HIC and LMIC participants.

"You know, we make meetings and they attend when they say they're going to, we do. So there's a certain amount of trust that you build up that way." ***(P4: Pharmacist, UK)***

For peer review only - http://bmjopen.bmj.com/site/about/guidelines.xhtml

Participants from HIC discuss the how LMIC found it 'refreshing' that HIC colleagues showed respect to colleagues in LMICs by not being forceful in their approach and thinking they know best.

"There was a comment from somebody from the one of the medical personnel in [redacted] that we met almost saying, oh, it's quite refreshing that you're not coming over here and telling us what we should be doing and we're like, well, no, that's not what we're here for, you know." ***(P13, Pharmacist, UK)***

Organised and accountable

Having individuals or small teams who were specifically responsible for ensuring the partnership was organised and kept to specific timelines, such as project managers, were described by both HIC and LMIC participants as vital for ensuring the partnership was delivered in an effective and timely manner. Partnership teams noticed the difference in performance when project managers were appointed in helping to deliver the project and tasks.

"[Project manager] was instrumental in organizing the Zoom meetings, and she has worked all round to ensure everything is going on well in the project." ***(P2: Pharmacist, Kenya)***

"I think we're very lucky with in [redacted] that we've got support from Global Health Partners, which are full time people who work to support parts of activity happening . . . They've been absolutely fabulous and trying to ensure that we stick the timetables". ***(P1: Microbiologist, UK)***

LMIC participants viewed it as important that project managers/steering committees were balanced and equal.

"So we have what we call the steering committee within our partnership . . . the committee was also based was also constituted visually equal equality. So you've got a balance across board." ***(P14: Pharmacist, Ghana)***

HIC participants discussed developing a work plan that was agreed by all, at the beginning of the specific project, was a useful tool to keep the partnership organised and accountable. This work plan also clearly highlighted expertise, roles and expectations, so the professional relationships and workload could be managed.

"We have a document with them which upholds exactly what we set out to achieve and we refresh that and we make sure we review that sort of time and time again, we'll probably be doing some again now post COVID. It definitely I think it shows a firm commitment and not just on a personal level, but actually an institutional commitment, which I think is really helpful in terms of getting the support and resources that you need behind the partnership." ***(P6: Pharmacist, UK)***

Responsible and build trust with partners

Building trust within partnerships was described as something that 'took time' and something that occurred due to familiarity and development of friendships. Having a face-

For peer review only - http://bmjopen.bmj.com/site/about/guidelines.xhtml

to-face connection and being able to spend time outside of work with colleagues helped to further develop trust was described by HIC participants.

"But having had that time together, you know not just in work, but also in a social context as well . . . you've got that kind of friendship element as well to it, which I think in the longer run is you know what we need in the partnership that sort of camaraderie and banter that comes with having had that sort of face-to-face interaction I think is important." **(P13: Pharmacist, UK)**

Whereas colleagues from LMICs talk about building trust from transparency and keeping each other accountable. They describe trust building each time a task has been completed from both LMIC and HIC collagues.

"For the whole project to be successive, each and everyone else have to do their part, so it created that mutual respect where no one feels like they're the ones doing more than the other party . . So over time and seeing the progress that we have been making, I think that helped us to devote that trust and that mutual respect." **(P11: Pharmacist, Malawai)**

Flexible, Resourceful and innovative

Allowing the partnership to be flexible, especially during the COVID-19 pandemic was important to allow everyone to contribute equally. Often things would change during the course of the partnership and participants discussed having to think innovatively about how to overcome certain obstacles.

"We had to have some contigency measures like due to one of them, or an example was when maybe flights were not possible to to be used. We have some contigency plans that we had, whereby one of them was the Zoom meetings." **(P2, Pharmacist, Kenya)**

"Yeah, and thinking innovatively and I think being flexible as well because it's not always time differences and stuff like that. It's not always easy as it as it seems, but you will make it happen." **(P6: Pharmacist, UK)**

Regular communication with partners and funders was expected, especially if changes needed to be made due to unexpected delays. This helped the partners continue to work towards their overall aim but within the boundaries of the partnership that were could often change quickly. Participants also discussed the importance of meeting regularly as a team, so no one had to make difficult decisions on their own.

"So when things come up, which we have to modify things, we notify each other as partners since we already in good contact then we also notify the Commonwealth Pharmacy Association and they were necessary it's because we got especially we need is to do with the change of our expenditure of money and also things that to do with the change of the protocols or change of the implementation plan of the project because we cannot just hold the goals." **(P11: Pharmacist, Malawi)**

For peer review only - http://bmjopen.bmj.com/site/about/guidelines.xhtml

Committed to joint learning

A core element was considered to be the continued learning that takes place between both countries. This often involved sharing experiences, tools and supporting each other with other issues outside of the specific aims of the partnership.

"The real key is that what it says the partnership, it's not about doing it one way, it's about it's about learning both ways. I think that we've learned so much from my [redacted] team as well. It's very much not being a one way thing." ***(P1: Microbiologist, UK)***

Learning was often perceived as a consequence of the partnerships but also encouraged throughout by sharing materials or mentoring people. This often went beyond the boundaries of the health partnerships to support people with their careers or preparation for similar issues such as the COVID-19 pandemic.

"I suppose, behind what we're doing, you know, is trying to say we're here to support, you know, because we've done this ourselves locally, it's trying to support you in doing the same thing, you know, in your context." ***(P13: Pharmacist, UK)***

Embed equity and inclusion

Ensuring the partnership foundations were based on equality, diversity and inclusion was considered a fundamental aspect to establishing the health partnership. This was sometimes formally monitored, with formal documents developed to ensure equality. These documents were signed when joining the health partnership, with consequences if breached. These ideas and formalisation of ideas often came from the LMIC participants.

"I think still based on the grounds of equality and diversity, we ensure, that's there's mutual respect and not just ensuring this. We've got documents that's back, whatever we do and there are sanctions for anyone that goes against the sort of offense, another party, so they, are laid down procedures to ensure that things are streamlined and properly formalized. Every member literally signed onto it. So I mean youv'e consented to abide by the rules, you go against it the loss would be with you." ***(P14: Pharmacist, Ghana)***

The focus and fundamental principle of equality appeared to drive flexibility within the partnership, by allowing the ability to be flexible and considerate of others.

"There's been that flexibility that has really helped, so that everyone is able to contribute equally do their tasks without yeah burdening one person or burdening few people." ***(P8: Project Manager, Malawi)***

A way in which the partnership ensured people were able to contribute equally in a practical sense was by using different platforms to communicate and edit documents.

"I think, using a platform like Google drive has helped us, and the weekly meetings have helped us to be able to share information equally and to share tasks equally. So I think that's basically how we've been doing it." ***(P8: Project Manager, Malawi)***

For peer review only - http://bmjopen.bmj.com/site/about/guidelines.xhtml

Recommendations for practice

To ensure this research promotes further effective practice in health partnerships, recommendations in line with the principles of partnerships have been developed, these are presented in Table 3.

For peer review only

For peer review only - http://bmjopen.bmj.com/site/about/guidelines.xhtml

Table 3: Recommendations for practice to support building a successful partnership

Principle	Example of enactment	Recommendation
Harmonised and aligned with national plans	*"Whatever we do, we try to consult the national policy documents in the national action plan so that at the end of the day, our actions are aligned . . . So you know there's always interconnectivity between whatever we are doing." **(P14: Pharmacist, Ghana)***	Guidance from the HIC, if relevant, should always be adapted for local use. Engaging with local stakeholders around how such guidance may work within the local context is good practice.
Effective and sustainable	*"There was a big Behavioural change, two of them on the team that came and so they helped with the activities . . . They shared with us, some modules and some metrics that we could use as we needed to continue with the project and actually if we were able to get the staff to accept it and to understand." **(P12: Project manager, Ghana)***	Experts in behavioural science if embedded in health partnerships multi-disciplinary teams can help to address behaviour change and long-term change. These experts can work on interventions and evaluations to drive and understand change.
Strategic	*"So it's maintaining that and sharing that bigger vision." **(P1: Microbiologist, UK)***	Health partnerships should focus upon developing a long-term, overarching vision and strategic aim(s). Whilst partnerships may focus upon small projects at a time, it is recommended that the health partnerships are formed as a partnership, rather than a project team. Showcasing the work within the partnerships can help to raise awareness of their benefits. Utilising case studies, writing up papers for publication and disseminating information is recommended.
Respectful and reciprocal	*"The relationships were being built on kind of basic based on kind of mutual respect and friendship really." **(P1: Microbiologist, UK)***	When setting up health partnerships, it is important to recruit individuals with the same values and vision. Carrying out informal interviews to understand their experience and values is recommended.
Organised and accountable	*"The massive gear change was halfway through the project. We had the project manager appointed." **(P13: Pharmacist, UK)***	Appointing a project manager and/or steering committee is recommended at the beginning of the project. Regular communication via platforms such as shared drives or messenger platforms which are acceptable for the team is recommended. This communication should be set out in a communication plan, so everyone is aware of when communication is expected but also how this communication with happen.

For peer review only - http://bmjopen.bmj.com/site/about/guidelines.xhtml

Responsible and build trust with partners	*"I think it's been built up over years. I wouldn't say it was just, you know, job done on day one kind of thing. I definitely think that's been cultivated over years and years." **(P6: Pharmacist, UK)***	Whilst participants discussed that health partnerships can work successfully without face-to-face visits, these are recommended where appropriate, as this can help to build rapport and trust between teams. Face-to-face visits also help teams gain a further understanding of the context, which can help to better develop interventions.
Flexible, resourceful and innovative	*"There's been that flexibility that has really helped, so that everyone is able to contribute equally do their tasks without burdening one person or burdening few people." **(P8: Project Manager, Malawi)***	Developing contracts to state expectations of how individuals should treat others and be treated, can help to eliminate any problems in ways of working and set boundaries within the partnership. Developing a work-plan with agreed time plan that allows for flexibility is recommended. This should be co-developed and regularly reviewed as a team.
Committed to joint learning	*"It's kind of this partnership of what we can share and the so much we've learned so much they've learned." **(P9: Pharmacist, UK)***	Including a bidirectional learning pathway in funding and timelines, where both teams share and learn from each other, could include visits/placements in both countries. Sharing CVs at the start of the project can help individuals identify mentors or expertise for further professional development.
Embed equity and inclusion	*"We do have a strategy which is gender equity or inclusion which we've decided to and incorporated into the process." **(P2: Pharmacist, Kenya)***	Health partnership embeding equity and inclusion is essential. Formalising this in the form of a strategy is recommended as this can ensure people are treated equally and supported appropriately as individuals. People can held people accountable if such strategies were breached. It also helps to set expectations for people who may join.

For peer review only

Discussion

This study identified how key principles of health partnerships are enacted within partnerships, specifically within CwPAMS, as an exemplar. Key findings of this study highlight the importance of good communication and transparency, the role of behavioural scientists/psychologists in ensuring sustainability and allowing all involved in the health partnership to have the opportunity to be heard and contribute fully.

This study supports previous findings, such as difficulties in communication, which include language barriers (17), or variations in language and behaviours amongst cultures (18) can be barriers to successful global health partnerships. Similar to findings by Jenkins and colleagues (2021), who found relationships that continue to flourish outside of work where friendships form, and shared appropriate language that invokes 'camaraderie' or 'banter' is fundamental in creating trust and transparency when building partnerships. While it may be important to monitor the frequency, type, and effectiveness of communications in order to evaluate partnership relationships (19), this would not take into account communication and relationships that occur outside of work in a social context. However, a communication plan detailing when and how meetings or updates will take place will help to reduce miscommunication between partners, aligning to both the *responsible* and the *respectful and reciprocal* Principles of Partnerships.

Behavioural scientists and psychologists were identified as playing a crucial role in the *Sustainability and effectiveness* of health partnerships. Previous research has identified the benefits of behavioural science within health partnerships (20, 21) and carious projects between the UK and Uganda, Sierra Leone, and Mozambique have demonstrated how behavioural scientists can help to strengthen global health partnerships whilst improving local health systems (4, 5). The Change Exchange (behavioural science volunteer programme) has aimed to bridge this gap by placing behavioural scientists in health partnerships including those in this study (21). As the partnerships all rely on behaviour change, whether that's behaviour change of clinical teams or communities to be successful, adding in experts in this area can help improve the effectiveness of the partnerships.

The COVID-19 pandemic has highlighted the importance of the *flexible*, *resourceful* and *innovative* principle. Partners have been forced to adapt to an ever-changing pandemic, therefore demonstrating openness and flexibility are fundamental to flourishing partnerships. Furthermore the COVID-19 pandemic disrupted in-person activities, whilst exacerbating exisiting inequalities in global health, especially in LMICs (22, 23). Teams had to quickly adapt and rely on virtual interactions and approaches (2). Research suggests there are differences between what LMIC and HIC partners value in virtual aspects of partnerships (2) and differences in how trust is established virtually compared to face-to-face (24). Having face-to-face contact has been associated with increased initial trust, compared to virtual, but over time, levels of trust are comparable between virtual and face-to-face teams (24). Establishing trust was viewed as a central component of partnerships, being able to have that human interation, coupled with each team completing tasks was found to increase trust.

For peer review only - http://bmjopen.bmj.com/site/about/guidelines.xhtml

Technology and shared drives have enabled individuals to contribute equally to discussions and documents. However, poor internet connections and conflicting daily priorities amongst institutions can cause difficulties in arranging meetings and document sharing between LMICs and HICs (2, 25). Thus, while individuals appear *committed to joint learning*, even during a global pandemic, it is important that the partnership considers the technological capacity within the partnership in order to maintain *respect* and *reciprocity* for all members of the partnership (2).

National governments in LMICs often change priorities for their own countries to meet the demands of international partners with their own agenda (26). For participants within this study, following local and national plans ensured partnerships were *harmonised* and *aligned*. Arguably, this gives individuals direction, and will ensure they are working towards the goal of the project without compromising their own governments' guidelines. It was acknowledged that UK guidelines cannot be implemented into LMICs without change; they must be altered to the local community whilst also utilising the skills of behavioural scientists and psychologists.

Critically, many studies have failed to include voices of all partners involved in global health partnerships, suggesting that, while many authors discuss equity, power relations are still prevalent (19, 27). Additionally, critical reflection about implementation to support the principles of partnership do not feature in much published work (28). This study attempted to overcome the issue of lack of representation by recruiting participants from both HICs and LMICs. However, this research is not without its limitations, interviews were carried out using an appreciative inquiry approach (13) and whilst this is a strength and allows the research team to explore common strengths in establishing and working in health partnerships, the barriers to enacting principles were less apparent. Further research should explore how behaviours within partnerships are enacted and what the influences on these behaviours are, to make a partnership 'work'. Further, the power relations are such a crucial part of partnerships that a robust exploration of power and its influence on enactment of partnership principles requires more attention. As identified above, partners critically reflecting on their partnerships is something that is important but often not studied. In this study, we did not specifically ask the partners whether they themselves spent time reflecting on the partnership and the enactment of the principles. A study of this reflection in and on partnership practice is warrented if we are understanding more deeply about what drives the enactment (or not) of principles. Another limitation is the interviews were conducted online due to participants being in different parts of the world. Carrying out the interviews online, whilst practically allowed for the research to be carried out, come with limitations as this can impact upon people's ability to be able to take part in the study due to requiring internet access and the appropriate devices. We did not present the findings and associated recommendations back to the participants or partnerships. This is a limitation, as it does not allow for grounding and checking with the participants, and should be a focus of future work.

For peer review only - http://bmjopen.bmj.com/site/about/guidelines.xhtml

Conclusion

Health partnerships are complex, with many different interacting parts. The nine Principles of Partnership developed by THET outline key elements to establish and develop a 'successful' partnership that goes beyond a partnership achieving its short-term project goals. Ten practical recommendations are suggested to support future health parnterships in enacting these principles, which include expectation setting from the inception of health partnerships, the role of behavioural scientists/psychologists in supporting health partnerships and continuing to highlight the importance of their work. Further work should continue to explore how to establish, sustained and evaluate health partnerships.

For peer review only

Contributorship

LBD and JH acquired the funding for this study. LBD, JH, EB and RT conceived the study design. RT and NC collected the data, with recruitment support from JF. RT led the qualitative analysis, with support from LBD, JH and EB. All authors revised the first manuscript draft and read and approved the final manuscript.

Acknowledgements

We would like to thank the study participants for their time and engagement in the study.

Patient and Public Involvement Statement

Patients and/or the public were not involved in the design, or conduct, or reporting, or dissemination plans of our research.

For peer review only

For peer review only - http://bmjopen.bmj.com/site/about/guidelines.xhtml

References

1. THET. The Tropical Health and Education Trust Health Partnership Scheme [Available from: https://www.thet.org/our-work/health-partnership-scheme/.
2. Umphrey L, Paasi G, Windsor W, Abongo G, Evert J, Haq H, et al. Perceived roles, benefits and barriers of virtual global health partnership initiatives: a cross-sectional exploratory study. Global Health Research and Policy. 2022;7(1):11.
3. Sneddon J, Thompson W, Kpobi LN, Ade DA, Sefah IA, Afriyie D, et al. Exploring the Use of Antibiotics for Dental Patients in a Middle-Income Country: Interviews with Clinicians in Two Ghanaian Hospitals. Antibiotics. 2022;11(8):1081.
4. Byrne-Davis L, Bull ER, Burton A, Dharni N, Gillison F, Maltinsky W, et al. How behavioural science can contribute to health partnerships: the case of The Change Exchange. Globalization and Health. 2017;13(1):30.
5. Bull ER, Mason C, Junior FD, Santos LV, Scott A, Ademokun D, et al. Developing nurse medication safety training in a health partnership in Mozambique using behavioural science. Global Health. 2017;13(1):45.
6. Plamondon KM, Brisbois B, Dubent L, Larson CP. Assessing how global health partnerships function: an equity-informed critical interpretive synthesis. Globalization and Health. 2021;17(1):73.
7. The Tropical Health and Education Trust. The Tropical Health and Education Trust: Principles of Partnership [Available from: https://www.thet.org/principles-of-partnership/.
8. Kulesa J, Brantuo NA. Barriers to decolonising educational partnerships in global health. BMJ Global Health. 2021;6(11):e006964.
9. Ackers HL, Ackers-Johnson J. Mobile professional voluntarism and international development: Killing me softly?: Springer Nature; 2017.
10. CwPAMS. [cited 2023 16th February]. Available from: https://commonwealthpharmacy.org/cwpams/.
11. Citrin D, Mehanni S, Acharya B, Wong L, Nirola I, Sherchan R, et al. Power, potential, and pitfalls in global health academic partnerships: review and reflections on an approach in Nepal. Glob Health Action. 2017;10(1):1367161.
12. Tong A, Sainsbury P, Craig J. Consolidated criteria for reporting qualitative research (COREQ): a 32-item checklist for interviews and focus groups. International journal for quality in health care. 2007;19(6):349-57.
13. Cooperrider DL, Srivastva, S. Research in organizational change and development 1987.
14. Cooperrider DL, Whitney D, Stavros JM. Appreciative inquiry handbook: For leaders of change, 2nd ed. San Francisco, CA, US: Berrett-Koehler Publishers; 2008. xxix, 454-xxix, p.
15. Malterud K, Siersma VD, Guassora AD. Sample Size in Qualitative Interview Studies:Guided by Information Power. Qualitative Health Research. 2016;26(13):1753-60.
16. Gale NK, Heath G, Cameron E, Rashid S, Redwood S. Using the framework method for the analysis of qualitative data in multi-disciplinary health research. BMC Med Res Methodol. 2013;13:117.
17. Hagen L, Munkhondya B, Myhre K. Similarities and mutual understanding: exchange experiences in Malawi for host and guest students. International Nursing Review. 2009;56(4):476-82.
18. Kokko R. Future nurses' cultural competencies: what are their learning experiences during exchange and studies abroad? A systematic literature review. J Nurs Manag. 2011;19(5):673-82.

For peer review only - http://bmjopen.bmj.com/site/about/guidelines.xhtml

19. Dean L, Njelesani J, Smith H, Bates I. Promoting sustainable research partnerships: a mixed-method evaluation of a United Kingdom–Africa capacity strengthening award scheme. Health Research Policy and Systems. 2015;13(1):81.
20. Turner RR, Reddy SE, Byrne-Davis L, Bull E, & , Hart J. An interview study to explore applied psychologists' experiences of implementing health psychology in global health partnerships: The Change Exchange. . British Journal of Health Psychology. 2023;In Press.
21. Byrne-Davis LMT, Bull ER, Burton A, Dharni N, Gillison F, Maltinsky W, et al. How behavioural science can contribute to health partnerships: the case of The Change Exchange. Globalization and Health. 2017;13(1):30.
22. Karim N, Rybarczyk MM, Jacquet GA, Pousson A, Aluisio AR, Bilal S, et al. COVID-19 pandemic prompts a paradigm shift in global emergency medicine: multidirectional education and remote collaboration. AEM Education and Training. 2021;5(1):79-90.
23. Shamasunder S, Holmes SM, Goronga T, Carrasco H, Katz E, Frankfurter R, et al. COVID-19 reveals weak health systems by design: why we must re-make global health in this historic moment. Global Public Health. 2020;15(7):1083-9.
24. Breuer C, Hüffmeier J, Hibben F, Hertel G. Trust in teams: A taxonomy of perceived trustworthiness factors and risk-taking behaviors in face-to-face and virtual teams. Human Relations. 2019;73(1):3-34.
25. Birch AP, Tuck J, Malata A, Gagnon AJ. Assessing global partnerships in graduate nursing. Nurse Educ Today. 2013;33(11):1288-94.
26. Schriger SH, Binagwaho A, Keetile M, Kerry V, Mubiligi JM, Ramogola-Masire D, et al. Hierarchy of qualities in global health partnerships: a path towards equity and sustainability. BMJ Glob Health. 2021;6(12).
27. Monette EM, McHugh D, Smith MJ, Canas E, Jabo N, Henley P, et al. Informing 'good' global health research partnerships: A scoping review of guiding principles. Global Health Action. 2021;14(1):1892308.

28. Bailie J, Laycock AF, Conte KP, et al. Principles guiding ethical research in a collaboration to strengthen Indigenous primary healthcare in Australia: learning from experience. BMJ Global Health 2021;6:e003852.

For peer review only

For peer review only - http://bmjopen.bmj.com/site/about/guidelines.xhtml

1

COREQ (COnsolidated criteria for REporting Qualitative research) Checklist

A checklist of items that should be included in reports of qualitative research. You must report the page number in your manuscript where you consider each of the items listed in this checklist. If you have not included this information, either revise your manuscript accordingly before submitting or note N/A.

Topic	Item No.	Guide Questions/Description	Reported on Page No.
**Domain 1: Research team and reflexivity**			
Personal characteristics			
Interviewer/facilitator	1	Which author/s conducted the interview or focus group?	8
Credentials	2	What were the researcher's credentials? E.g. PhD, MD	n/a
Occupation	3	What was their occupation at the time of the study?	n/a
Gender	4	Was the researcher male or female?	n/a
Experience and training	5	What experience or training did the researcher have?	n/a
Relationship with participants			
Relationship established	6	Was a relationship established prior to study commencement?	8
Participant knowledge of the interviewer	7	What did the participants know about the researcher? e.g. personal goals, reasons for doing the research	n/a
Interviewer characteristics	8	What characteristics were reported about the inter viewer/facilitator? e.g. Bias, assumptions, reasons and interests in the research topic	n/a
**Domain 2: Study design**			
Theoretical framework			
Methodological orientation and Theory	9	What methodological orientation was stated to underpin the study? e.g. grounded theory, discourse analysis, ethnography, phenomenology, content analysis	8
Participant selection			
Sampling	10	How were participants selected? e.g. purposive, convenience, consecutive, snowball	8
Method of approach	11	How were participants approached? e.g. face-to-face, telephone, mail, email	8
Sample size	12	How many participants were in the study?	9
Non-participation	13	How many people refused to participate or dropped out? Reasons?	n/a
Setting			
Setting of data collection	14	Where was the data collected? e.g. home, clinic, workplace	8
Presence of non-participants	15	Was anyone else present besides the participants and researchers?	8
Description of sample	16	What are the important characteristics of the sample? e.g. demographic data, date	8
Data collection			
Interview guide	17	Were questions, prompts, guides provided by the authors? Was it pilot tested?	8
Repeat interviews	18	Were repeat inter views carried out? If yes, how many?	n/a
Audio/visual recording	19	Did the research use audio or visual recording to collect the data?	8
Field notes	20	Were field notes made during and/or after the inter view or focus group?	8
Duration	21	What was the duration of the inter views or focus group?	8
Data saturation	22	Was data saturation discussed?	8
Transcripts returned	23	Were transcripts returned to participants for comment and/or	n/a

2

Topic	Item No.	Guide Questions/Description	Reported on Page No.
		correction?	
**Domain 3: analysis and findings**			
Data analysis			
Number of data coders	24	How many data coders coded the data?	8-9
Description of the coding tree	25	Did authors provide a description of the coding tree?	n/a
Derivation of themes	26	Were themes identified in advance or derived from the data?	9
Software	27	What software, if applicable, was used to manage the data?	8-9
Participant checking	28	Did participants provide feedback on the findings?	n/a
Reporting			
Quotations presented	29	Were participant quotations presented to illustrate the themes/findings? Was each quotation identified? e.g. participant number	9-18
Data and findings consistent	30	Was there consistency between the data presented and the findings?	9-18
Clarity of major themes	31	Were major themes clearly presented in the findings?	9-18
Clarity of minor themes	32	Is there a description of diverse cases or discussion of minor themes?	9-18

Developed from: Tong A, Sainsbury P, Craig J. Consolidated criteria for reporting qualitative research (COREQ): a 32-item checklist for interviews and focus groups. *International Journal for Quality in Health Care*. 2007. Volume 19, Number 6: pp. 349 – 357

Once you have completed this checklist, please save a copy and upload it as part of your submission. DO NOT include this checklist as part of the main manuscript document. It must be uploaded as a separate file.

MANCHES 182
The University of Ma

3

Understanding Principles of Partnerships for Commonwealth Antimicrobial Stewardship Schemes

Semi-structured interview schedule

Introduction

Thank you for your time in taking part today. We are interested in your perspective regarding the delivery of key principles of partnerships and what makes health partnerships successful. Just to reiterate there is no right or wrong answer and we are simply interested in your opinion. The interview today and the data generated in the form of transcript will be kept within the research team and any identifiable information or experiences shared will be fully anonymised. No identifiable details will be shared and only general points will be fed back to THET. Do you have any questions?

Before we start, let me explain a bit about what we are going to do, because it may be a little different from what you have done in the past. This will be what's called an 'appreciative interview'. I will be asking you questions about when you have experienced things working at their best and what made it happen that way, because the more we know what makes things work well, the more we can use these successful elements in the future. I won't be asking about problems or searching for ways to fix them. Instead, I would like to hear about what is working well, and the factors that make things work well. Do you have any questions?

Questions

Can you tell me a little bit about the health partnership you are/were involved in?
Probe: Project, aims of project, countries involved.

- Does you project or previous project explore an aspects of behaviour change for antimicrobial stewardship?
    - Probe: How is this being measured, how is this being addressed?

- What behavioural strategies have you found work well in your partnerships?
    - Probe: acceptability

What's really important about this experience? What do you value most about it?

Thinking about the partnership aspects of your work, what do you think has gone well?

What key strengths do you think have been important in this work?

What have you or your partners done well to try to make your partnership strategic?
Probe: shared vision, long-term aims, agreed priorities

For peer review only - http://bmjopen.bmj.com/site/about/guidelines.xhtml
For peer review only

What have you or your partners done well to make you partnership aligned to national plans?

What have you or your partners done well to make your partnership meet your target and aims?
What have you or your partners done well to make your partnership reciprocal and respectful?

What have you or your partners done well to make your partnership ‘equal’ or ‘shared’?

What have you or your partners done well to make your partnership organised and accountable?
Probe: Structure, management, transparent, clear

What have you or your partners done well to cultivate trust with partners or other stakeholders?

Where you’ve experienced changes during your partnership, what have you or your partners done well to make your partnership flexible and innovative in the face of change?

What have you or your partners done well to continue learning together?

What have you or your partners done well to tackle inequalities in your partnership?

Can I ask you to imagine yourself in a year or two years in the future, where the partnership has worked really well.
Probe: What does that look like?
Probe: What has happened?
Probe: How did you make that work?
Probe: What support would you need to achieve this?

Is there anything else you wish to say or discuss today?

For peer review only

For peer review only - http://bmjopen.bmj.com/site/about/guidelines.xhtml
